# Injectable In Situ Thermoreversible Gel Depot System of Lidocaine Nanoemulsion for Prolonged Anesthetic Activity in Dental and Operative Procedures

**DOI:** 10.3390/pharmaceutics17101355

**Published:** 2025-10-20

**Authors:** Shery Jacob, Fathima Sheik Kather, Shakta Mani Satyam, Sai H. S. Boddu, Firas Assaf, Tasnem H. Abdelfattah Allam, Anroop B. Nair

**Affiliations:** 1 Department of Pharmaceutical Sciences, College of Pharmacy, Gulf Medical University, Ajman P.O. Box 4184, United Arab Emirates; fathima.sheik@gmu.ac.ae; 2 Department of Pharmaceutics, RAK College of Pharmacy, RAK Medical and Health Sciences University, Ras Al Khaimah P.O. Box 11172, United Arab Emirates; satyam@rakmhsu.ac.ae (S.M.S.); firas.22901067@rakmhsu.ac.ae (F.A.); 3 Department of Pharmaceutical Sciences, College of Pharmacy and Health Sciences, Ajman University, Ajman P.O. Box 346, United Arab Emirates; s.boddu@ajman.ac.ae (S.H.S.B.); 202110187@ajmanuni.ac.ae (T.H.A.A.); 4 Center of Medical and Bio-Allied Health Sciences Research, Ajman University, Ajman P.O. Box 346, United Arab Emirates; 5 Department of Pharmaceutical Sciences, College of Clinical Pharmacy, King Faisal University, Al-Ahsa 31982, Saudi Arabia; anair@kfu.edu.sa

**Keywords:** local anesthesia, lidocaine, nanoemulsion, nanoemulgel, characterization, in vivo studies

## Abstract

**Background/Objectives:** Lidocaine hydrochloride (LD-HCl) is the most commonly used local anesthetic in dentistry, often administered with epinephrine to extend its duration and reduce systemic absorption. However, its relatively short duration of action, the need for repeated injections, and the unpleasant taste may limit patient compliance and procedural efficiency. This study aimed to develop and evaluate a novel injectable nanoemulsion-based in situ gel depot system of LD to provide prolonged anesthetic activity. **Methods:** LD-loaded nanoemulsions were formulated by high-shear homogenization followed by probe sonication, employing Miglyol 812 N (oil phase), a combination of Tween 80 and soy lecithin (surfactant–co-surfactant), glycerin, and deionized water (aqueous phase). The selected nanoemulsion (S1) was dispersed in a thermoreversible poloxamer solution to form a nanoemulgel. The preparation was evaluated for globule diameter and uniformity, zeta potential, surface morphology, pH, drug content, stability, rheological behavior, injectability, and in vitro drug release. Analgesic efficacy was assessed via tail-flick and thermal paw withdrawal latency tests in Wistar rats. Cardiovascular safety was monitored using non-invasive electrocardiography and blood pressure measurements. **Results:** The developed nanoemulsions demonstrated a spherical shape, nanometer size (206 nm), high zeta-potential (−66.67 mV) and uniform size distribution, with a polydispersity index of approximately 0.40, while the nanoemulgel demonstrated appropriate thixotropic properties for parenteral administration. In vitro release profiles showed steady LD release (5 h), following the Higuchi model. In vivo studies showed significantly prolonged analgesic effects lasting up to 150 min (2.5 h) compared to standard LD-HCl injection (*p* < 0.001), with no adverse cardiovascular effects observed. **Conclusions:** The developed injectable LD in situ nanoemulgel offers a promising, patient-friendly alternative for prolonged anesthetic delivery in dental and operative procedures, potentially reducing the need for repeated injections and enhancing procedural comfort.

## 1. Introduction

Local anesthetics (LAs) often have a short duration of action and can cause local or systemic toxicity. Lidocaine (LD) exerts its effect by reversibly blocking nerve impulse conduction, thereby preventing the propagation of nociceptive signals towards the central nervous system. Approaches to improve their efficacy and safety include synthesizing or enantioseparating new LA molecules, adjusting pH, combining them with analgesics such as opioids, and developing advanced drug delivery systems [1,2]. Among the various LAs, lidocaine hydrochloride (LD HCl) is a rapidly acting drug extensively employed in dental practice and surgical procedures owing to its safety, efficacy, and tolerability. A 2% LD HCl solution combined with epinephrine bitartrate (1:50,000 or 1:100,000), a potent vasoconstrictor, is commonly administered (0.5–2 mL) to achieve effective and prolonged analgesia. LD remains preferred over longer-acting anesthetics (e.g., bupivacaine) in many settings because of its more favorable safety profile, particularly in pregnancy (FDA category B), better tolerability, and lower incidence of injection pain [3,4]. However, challenges such as reflux of solution into the oral cavity, often due to patient movement or poor technique, can result in patients tasting the bitter formulation and experiencing systemic effects from epinephrine, such as jitteriness and transient palpitations. Similarly, in certain operative or outpatient surgical procedures, repeated infiltration of LD may be necessary to maintain adequate anesthesia, which can be inconvenient for both patients and clinicians.

To prolong anesthetic duration, various sustained-release systems have been developed. Hydrogels have emerged as biocompatible systems capable of slowing drug clearance and prolonging anesthetic duration. Poloxamers are nonionic, synthetic triblock copolymers of polyethylene oxide (PEO) and polypropylene oxide (PPO) widely explored in drug delivery for enhancing drug biopharmaceutics, pharmacodynamics, and pharmacokinetics. Poloxamer-based gels are known for their biocompatibility and biodegradability, reducing the risk of tissue irritation, clogging, or chronic inflammation at the injection site, a key consideration for any long-acting depot formulation [5]. Their key advantage lies in thermoreversible gelation near body temperature, driven by micellization. Similarly, an LD-loaded microsphere-poloxamer 407 gel system demonstrated a markedly prolonged sensory and motor block compared to regular formulations, emphasizing its potential for effective postoperative pain management [6]. Additionally, this gel system provides sustained LD release, ensuring prolonged anesthesia while minimizing systemic toxicity in clinical applications. However, these systems often suffer from slower onset, higher viscosity, and reduced injectability, which may hinder their use in fine infiltration or limit perineural spread. Thus, an injectable depot formulation based on a thermoreversible hydrogel has the potential to extend the duration of analgesia with a single administration, thereby minimizing dosing frequency while enhancing procedural efficiency and patient comfort [7,8].

Encapsulation of drugs within lipid nanocarriers represents a promising strategy for efficient and sustained delivery, offering controlled physicochemical properties that enhance bioavailability while minimizing the likelihood of adverse effects [9]. Within the spectrum of lipid-based nanosystems, nanoemulsions are considered the preferred option for parenteral drug delivery because of their distinctive features and superior benefits relative to other lipid nanoparticles [10]. For instance, intravenously administered total parenteral nutrition lipid emulsions are typically formulated as oil-in-water (o/w) systems having a typical particle size of below 500 nm, as specified by pharmacopeial standards such as the United States Pharmacopeia and European Pharmacopoeia. When prepared and administered according to regulatory guidelines, these emulsions are considered safe, well-tolerated, and effective for long-term parenteral feeding in patients unable to receive adequate nutrition by oral or enteral routes [11]. Preclinical studies have demonstrated that formulation architecture influences vascular compatibility, as shown in comparative evaluations of lipid emulsions and microemulsions [12]. In addition, recent reviews on injectable biomaterials underscore the critical importance of vascular safety, emphasizing hemocompatibility and inflammatory response evaluations as essential steps for their safe clinical translation [13].

LD is classified as a Class II drug in the Biopharmaceutics Classification System, exhibiting limited aqueous solubility but favorable membrane permeability [14]. Its poor water solubility can limit formulation options, making it a suitable candidate for lipid-based drug delivery systems such as nanoemulsions. LD’s physicochemical properties, including a log *p* value of 2.26, a relatively low molecular weight of 234.34 g/mol, and a low therapeutic dose, render it an ideal candidate for formulation into nanoemulsions intended for parenteral delivery [15].

Nanoemulsions can enhance the solubilization of lipophilic drugs like LD, providing a homogenous dispersion in biocompatible oils that allows for controlled drug release and rapid onset of action. Additionally, nanoemulsions intended for parenteral administration can be designed with droplet sizes near 200 nm, ensuring high physical stability, reduced injection pain, and minimized risk of embolism [16]. Injectable nanoemulsions, while effective in improving the solubility and dispersion of poorly water-soluble drugs such as LD, generally fail to provide prolonged local retention, as the drug can rapidly partition into surrounding tissues and undergo systemic clearance. To overcome this limitation, the incorporation of nanoemulsions into a thermoresponsive gel matrix offers a promising strategy. LD-loaded nanoemulsions offer rapid onset, improving permeation, and lower viscosity, thermoreversible gelation by poloxamer offers localized retention and sustained release. By combining both, the nanoemulsion-in-gel system aims to achieve onset kinetics closer to a standard LD injection while extending duration into a clinically useful window. Upon administration, the formulation transitions from a free-flowing sol to a viscoelastic gel, reducing drug clearance, prolonging anesthetic action, and minimizing systemic toxicity. The nanoemulsion droplets remain uniformly distributed within the gel matrix, reducing their mobility, ensuring consistent drug release and maintaining physical stability during application. This dual functionality of enhancing solubility and simultaneously extending local drug retention provides a clear advantage of nanoemulgels over conventional nanoemulsions for injectable anesthetic delivery [17].

This study aims to design and characterize an injectable LD nanoemulsion embedded within an in situ thermoreversible gel depot to prolong local anesthesia during dental and operative procedures. All excipients utilized in the present study, including the oil, surfactant, co-surfactant, and gelling agents, are GRAS-certified and approved for parenteral use in accordance with FDA and EMA guidelines. Safety concerns for injectable nanoemulsions and nanoemulgels have been addressed in line with established regulatory guidance [18,19]. The nanoemulsion-in-gel system was specifically designed to achieve a ~150-min release, a duration sufficient to cover common outpatient procedures such as complex dental extractions, root canal therapy, periodontal surgeries, laparoscopic port-site infiltration, minor anorectal or cystoscopic interventions, and dermatologic procedures without the need for reinjection or transition to longer-acting anesthetics. In contrast, conventional LD-HCl typically provides only 60–90 min of anesthesia, which often wears off mid-procedure or during the immediate postoperative period, necessitating supplemental dosing [5,20,21]. The research scope includes formulation development of nanoemulsions using FDA-approved oils, surfactants, and cosurfactants; comprehensive physicochemical characterization; and preclinical evaluation of the LD-loaded gel in Wistar rats. The objectives are to formulate LD nanoemulsions, incorporate the selected nanoformulation into a thermoreversible poloxamer gel to assess gelation behavior and injectability, evaluate in vitro release and stability, and determine analgesic efficacy using tail-flick and thermal paw-withdrawal tests along with cardiovascular safety through non-invasive ECG and blood pressure monitoring. The working hypothesis is that the LD nanoemulsion-in-gel depot will extend the duration of analgesia while maintaining acceptable cardiovascular safety in vivo. The approach involves sequential formulation and characterization followed by animal testing to evaluate analgesic performance and cardiovascular safety using established procedures. Though the development of a hybrid system is more complex than simple depots, the anticipated clinical advantage in terms of fewer injections, continuous analgesia, procedural efficiency, and better patient comfort justifies the complexity.

## 2. Materials and Methods

### 2.1. Materials

LD was purchased from Sigma-Aldrich (Darmstadt, Germany). Miglyol 812 N was kindly donated by IOI Oleo Chemicals (Hamburg, Germany). Poloxamer 407 was purchased from Sigma-Aldrich (Darmstadt, Germany); Soy lecithin (30%) and Tween 80 were procured from SRL Chem (Mumbai, India). Acetonitrile (99.98%) and HPLC-grade water were obtained from EuroLab (Middlesex, UK). All other chemicals that were of reagent grade were used as received.

### 2.2. HPLC Analysis of Lidocaine

LD content in the different nanoemulsion formulations was analyzed using a Shimadzu HPLC apparatus (Kyoto, Japan) and Phenomenex Luna C18 column (150 × 4.6 mm, 5 μm, 100 Å) maintained at 40 °C. The device consists of pump (LC-20AD), degasser (DGU-20A3), detector (SPD-20A UV–VIS). The mobile phase was delivered at a flow rate of 1.5 mL/min and consisted of 20 mM phosphate buffer (pH 5.5) combined with acetonitrile in a 74:26 (*v*/*v*) ratio. A 10 µL sample was injected, and LD detection was carried out at 273 nm.

### 2.3. Screening of Oil

To determine the solubility of LD in various oils, a vortex mixer was used to mix an excess amount of the drug with 2 mL of each oil (Miglyol^®^ 812 N, Miglyol^®^ 829, Miglyol^®^ 840, castor oil, and triacetin) in separate 5 mL stoppered glass vials. The samples were kept at room temperature (25 ± 1 °C) for 72 h at 180 rpm on an isothermal shaker (Incu-Shaker, Benchmark Scientific, Edison, NJ, USA). Following 15 min centrifugation at 3000 rpm, the supernatant was extracted and passed through membrane filter (pore size 0.22 µm). Data are expressed as mean ± S.D., calculated from three replicates for each oil sample. The LD content in each individual sample was then quantified using HPLC.

### 2.4. Selection of Surfactants and Co-Surfactants

Similar to oil screening, LD’s solubility in various surfactants, including Tween 80, Tween 20, and soy lecithin, was assessed. For solubility assessment, surfactants and co-surfactants were evaluated in their pure form at room temperature. Data are expressed as mean ± S.D., based on triplicate measurements obtained for each surfactant and co-surfactant sample.

### 2.5. Preparation of Lidocaine-Loaded Nanoemulsion

**Figure 1:**
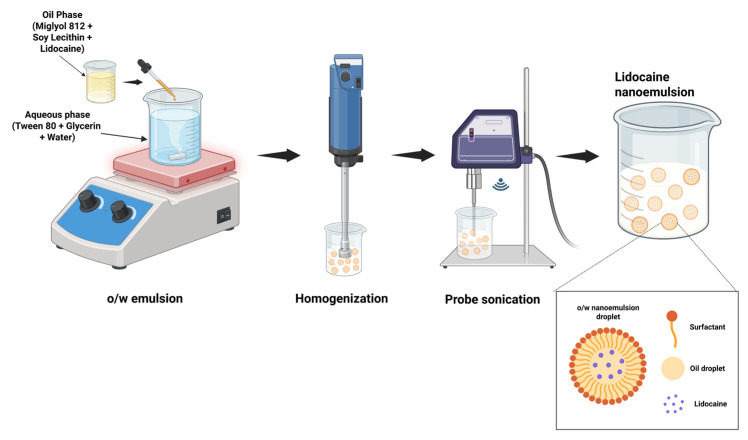
Schematic representation of the method used for preparing lidocaine-loaded nanoemulsion.

Figure 1 illustrates the schematic procedure used for nanoemulsion preparation. The preparation of the injectable LD nanoemulsion begins with the selection of suitable components, including Miglyol 812 N (7.5% *w*/*v*) as the oil phase, glycerin (2.25% *w*/*w*) and distilled water as the aqueous phase, and Tween 80 (1% *w*/*v*) with soy lecithin (3% *w*/*v*) as a surfactant and co-surfactant, respectively. LD (2% *w*/*v*) was first dissolved in Miglyol 812 N and soy lecithin by gentle mixing, followed by heating to 60–75 °C to ensure complete solubilization. In parallel, the aqueous phase, consisting of Tween 80 and glycerin incorporated to improve viscosity, ensure isotonicity, and enhance the stability of the formulation, was heated to the same temperature of the oil phase, thereby minimizing the risk of phase separation. All aqueous components of the formulation were prepared using pyrogen-free sterile water for injection, and all glassware and stainless-steel instruments were depyrogenated at 250 °C for 30 min before use. The oil phase was then added dropwise to the aqueous phase under continuous stirring at 1000–1200 rpm using a magnetic stirrer (DLAB, MS-H380 Pro, Beijing, China) for 15–20 min to achieve primary emulsification. The resulting o/w emulsion was subjected to high-shear homogenization employing an Ultra-Turrax T25 homogenizer (IKA Werke, Staufen, Germany) at 8000–10,000 rpm for approximately 15 min to reduce droplet size and ensure uniformity. Finally, the nanoemulsion was further processed using a programmable probe sonicator (QSonica Q125-110, Newtown, CT, USA; maximum output power 125 W). Sonication was performed at 30% amplitude (corresponding to ~37.5 W) in pulse mode, with 5 s ON/5 s OFF cycles, for a total processing time of 5 min. All excipients used in the current formulation complied with USP/NF endotoxin limits, as verified by their certificates of analysis. Future studies should evaluate and ensure that the endotoxin level remains below the acceptable limit (<0.25 EU/mL) specified for parenteral preparations in the final nanoemulsion using the Limulus Amebocyte Lysate (LAL) assay. All studies were conducted in triplicate, and results are reported as mean ± standard deviation (S.D.).

### 2.6. Characterization of LD-Loaded Nanoemulsion

#### 2.6.1. Drug Content

Quantification of LD content was carried out by transferring a precisely measured 1 mL aliquot of the LD-loaded nanoemulsion to a polypropylene centrifuge tube, followed by the addition of 9 mL of mobile phase (added in portions with gentle mixing to ensure complete extraction of the drug). The mixture was shaken for 1 h using a mechanical shaker (Incu-Shaker, Benchmark Scientific, Edison, NJ, USA) to achieve thorough dispersion and drug release from the emulsion matrix. Samples were then centrifuged (MiniSpin centrifuge, Eppendorf, Germany) at 4280× *g* for 10 min to separate the clear supernatant from the emulsion droplets. The supernatant was filtered through a 0.22 µm syringe filter, and 1 mL of the filtrate was collected and appropriately diluted for HPLC analysis.

Entrapment efficiency (EE%) was determined by ultracentrifugation to separate free drug from the nanoemulsion. Briefly, 0.5 mL of dispersion was ultracentrifuged (20,000× *g*, 20 min, 25 °C) and the supernatant was quantified by a previously described HPLC assay. EE% was calculated as (C_total_ − C_free_)/C_total_ × 100.

Drug loading (DL%) was calculated from the entrapped drug content obtained in the EE study relative to the total solid mass of the formulation after lyophilization (Labconco, Kansas, MO, USA) using the equation: (mass of drug entrapped/mass of total solids) × 100.

#### 2.6.2. Percentage Transmittance and pH

The pH of LD-loaded nanoemulsions was determined at 25 ± 1 °C using a pre-calibrated pH meter (Jenway 3510, Staffordshire, UK). Optical clarity was evaluated by determining transmittance with a UV spectrophotometer (Shimadzu, Kyoto, Japan). For instrument calibration, a blank cuvette containing double-distilled water was used to establish 100% transmittance at 400 nm. The selected formulations were diluted 25-fold with water prior to analysis, and percentage transmittance values were recorded.

#### 2.6.3. Dilution Potential

A dilution study was carried out to assess the likelihood of phase inversion in the selected nanoemulsions. In this procedure, 1 mL of the nanoemulsion was diluted tenfold using deionized water and observed for any evidence of phase separation.

#### 2.6.4. Particle Size and Zeta (ζ) Potential

The size, polydispersity index (PDI), and ζ potential of the developed formulations were determined using a Horiba Zetasizer (Model SZ-100, Kyoto, Japan) at a scattering angle of 90°. Briefly, 1 mL of each test sample was loaded into the analysis chamber and assessed using the Horiba SZ-100 software (Z-type, version 2.20, Horiba, Japan) [22].

#### 2.6.5. Viscosity

An Atago viscometer (Visco-895, Tokyo, Japan) was used to test the viscosity of the selected LD-loaded nanoemulsions at 25 °C over a range of angular velocities [19].

#### 2.6.6. Kinetic Stability Studies

To assess the physical stability of the selected nanoemulsion formulation, a series of stress tests was conducted following standard protocols [23]. A heating–cooling stability test was performed to replicate temperature fluctuations, consisting of six consecutive cycles between 4 ± 1 °C and 45 ± 1 °C, with each temperature maintained for a minimum of 48 h. The study was carried out using a domestic fridge (LG Electronics, Delhi, India) and an oven with hot air (SCT-Convect-1, Sci-Chem, Mumbai, India) to simulate storage under extreme climatic conditions. Centrifugation stress testing was performed to detect possible phase separation by subjecting the nanoemulsions to 3500 rpm for 30 min using a high-speed centrifuge (MiniSpin, Eppendorf, Germany), as previously recommended for accelerated stability screening. Furthermore, a freeze–thaw cycle test was conducted by subjecting the formulations to three cycles of alternating temperatures between −21 ± 1 °C (deep freezer; Forma, Thermo Scientific, Waltham, MA, USA) and 25 ± 1 °C (ambient temperature), with each phase maintained for at least 48 h.

#### 2.6.7. Transmission Electron Microscopy (TEM)

TEM was used to examine the vesicle shape using a TFS TALOS F200X (Waltham, MA, USA) device running at 200 kV. For sample preparation, a drop of the nanoemulsion was placed on a carbon-coated copper grid and allowed to adsorb for 10 s. The grid was subsequently rinsed gently with deionized water in multiple washes (25 times) and air-dried at ambient temperature (25 ± 2 °C). Once dried, the samples were visualized under TEM using bright-field imaging at progressively higher magnifications to capture high-resolution images of the nanoemulsion droplets.

### 2.7. Preparation of Lidocaine Loaded Nanoemulgel

Poloxamer 407 was gradually dispersed in deionized water to prepare a 40% *w*/*w* stock solution, which was then hydrated under refrigerated conditions (4 ± 1 °C) for 24 h to ensure complete polymer swelling and solubilization. To prepare the final in situ nanoemulgel, 0.9 mL of the selected LD nanoemulsion (containing 2% *w*/*v* LD base) was homogeneously blended with 0.6 mL of the prehydrated poloxamer solution, resulting in a final Poloxamer 407 concentration of 18% *w/v* and stored at 2–8 °C. The formulation was calibrated such that each 1.5 mL dose of the nanoemulgel contains 18 mg of LD base, equivalent to 20.8 mg of LD HCl, ensuring accurate dosing suitable for parenteral administration. All formulation steps, including preparation of the selected nanoemulsion and the final nanoemulgel, were performed under aseptic conditions in a class II, Type A2 biosafety cabinet (Labconco, Kansas City, MO, USA). Sterilization of thermoresponsive nanoemulgel systems remains challenging, as conventional methods may compromise droplet stability or alter the sol–gel transition, a limitation also noted in recent literature [24].

### 2.8. Characterization of Nanoemulgel

A visual inspection of the LD-loaded nanoemulgel was carried out to check for appearance, consistency, and phase stability.

#### 2.8.1. pH and Drug Content

Quantitative determination of LD content in the nanoemulgel formulations was conducted using a validated HPLC method, as described previously. Briefly, a known volume (1 mL) of each nanoemulgel sample was accurately withdrawn and diluted with an appropriate volume of the mobile phase to disrupt the gel matrix and facilitate complete drug extraction. The mixture was subjected to mechanical agitation utilizing a vortex mixer (REMI CM-101, Mumbai, India) for 30–60 min, followed by centrifugation at 4000–5000× *g* (MiniSpin, Eppendorf, Hamburg, Germany) for 10–15 min to remove any undissolved excipients or particulates. Subsequently, the clarified supernatant was filtered using a 0.22 μm syringe filter before being subjected to HPLC analysis. The pH of LD-loaded gel formulations was measured with a calibrated pH meter (Jenway 3510, Staffordshire, UK) by immersing the electrode into the gel and allowing it to stabilize for 1 min.

#### 2.8.2. Viscosity

The viscosity of the LD-loaded thermosensitive nanoemulgel was determined using a rotational viscometer (Atago VISCO™, Atago Co., Ltd., Tokyo, Japan) equipped with an appropriate spindle (L-2) suitable for semi-solid and gel formulations. Measurements were conducted at 37 ± 0.5 °C to simulate physiological conditions, using a temperature-controlled sample chamber to ensure thermal stability throughout the test [25]. Approximately 20 mL of the nanoemulgel formulation was carefully transferred into the viscometer cup, ensuring the absence of air bubbles. The apparent viscosity, expressed in cP, was recorded over a range of controlled shear rates by varying the rotational speed from 250 rpm to 50 rpm in a descending mode to evaluate shear-thinning behavior, which is characteristic of poloxamer-based systems. Each reading was allowed to reach equilibrium before data acquisition, and a flow curve (viscosity vs. shear rate) was constructed to assess the pseudoplastic nature of the formulation. All rheological measurements were performed in triplicate, and the mean values were reported with standard deviations.

#### 2.8.3. Sol–Gel Transition and Injectability Testing

The in situ gelation or sol–gel transition of the nanoemulgel was evaluated using the tube inversion method with minor modifications [26]. Approximately 2 mL of the sol formulation, previously stored at 2–8 °C, was placed in a test tube and immersed in a water bath set at 37 ± 0.5 °C. The tube was tilted at a 90° angle, and gelation was confirmed when the meniscus remained stationary upon tilting. The time required for gel formation was recorded accordingly.

The injectability of the nanoemulgel formulation was quantitatively evaluated using a Brookfield CT3 50K Texture Analyzer (AMETEK Brookfield, Middleborough, MA, USA) operated in compression mode. The test was designed to simulate manual administration and determine the extrusion force required for delivering the formulation through two different syringe-needle systems: 26-gauge (26 G) and 23-gauge (23 G) [27]. Approximately 0.1 mL of the nanoemulgel was loaded into standard 1 mL plastic syringes at room temperature (25 ± 1 °C), which were mounted vertically on the instrument’s testing platform. The plunger of each syringe was aligned beneath a stainless-steel cylindrical TA5 probe. The texture analyzer settings were as follows: pre-test speed of 2.00 mm/s, test speed of 0.50 mm/s, and post-test (return) speed of 10.00 mm/s. A trigger force of 0.100 N was set to initiate compression, with a hold time of 5 s at the maximum compression point. Force-displacement data were acquired at a sampling rate of 10.00 points/s. A 1000 g load cell (equivalent to ~9.8 N) was used for force detection, and each sample was subjected to a single compression cycle. The maximum force in Newtons required to extrude the formulation through each needle size was recorded.

### 2.9. In Vitro Release

The in vitro release of LD from the nanoemulgel formulation was investigated using a dynamic dialysis method, adapted for semisolid and nanoformulated drug delivery systems [28]. A pre-activated cellophane dialysis membrane (molecular weight cut-off: 12–14 kDa), previously soaked in deionized water for 24 h, was used as the diffusion barrier. Weighed quantity of nanoemulgel (1 g) or solution (1.35 mL) (containing 12 mg pure LD base) was carefully placed inside a dialysis tubing segment, which was sealed at both ends and immersed in 250 mL of phosphate-buffered saline (PBS, pH 7.4) containing 0.05% Tween 80 to maintain sink conditions. The entire setup was controlled at 37 ± 0.5 °C in order to mimic physiological conditions. Continuous agitation at 250 rpm was applied using a magnetic stirrer to enhance diffusion across the membrane. At predetermined intervals (1, 2, 3, 4, and 5 h), 1 mL samples were withdrawn from the release medium and replenished with an equal volume of pre-warmed buffer to preserve sink conditions. The collected samples were diluted with the mobile phase as required and analyzed for drug concentration using a validated HPLC method. Drug release profiles were analyzed using kinetic modeling by fitting the data to various mathematical models, including zero-order, first-order, Higuchi, and Korsmeyer–Peppas equations, to elucidate the release mechanism [29].

### 2.10. Stability Assessment

Stability testing of the best-performing LD-loaded nanoemulsion was carried out over three months under refrigerated conditions (2–8 °C) using amber containers. Parameters such as PDI, ζ potential, droplet size, dilution behavior, drug content (%), pH, transmittance (%), phase separation, flocculation, precipitation, and viscosity were monitored [30]. For three months, the LD-loaded nanoemulgel was simultaneously kept in glass vials in a climate chamber (Memmert, Schwabach, Germany) at a controlled temperature of 25 ± 0.2 °C and 75 ± 5% relative humidity [31]. At scheduled time points, samples were withdrawn and examined for visual appearance, pH, viscosity, and drug content.

### 2.11. Animal Studies

Male Wistar rats (Rattus norvegicus) aged 8–10 weeks and weighing 250–320 g were used in the study. All in vivo studies were conducted in accordance with institutional and international ethical standards for animal research. The experimental procedures were conducted in accordance with the protocol that Ras Al Khaimah Medical and Health Sciences University’s Animal Ethics Committee (AEC) has authorized (AEC-RAKMHSU-PG-C-01-2024-2025). The study adhered to the National Institutes of Health (NIH) Guide for the Care and Use of Laboratory Animals (NIH Publication No. 85-23, revised 1985), which provides internationally accepted guidelines for the ethical and humane treatment of laboratory animals [32]. Standard laboratory food and water were freely available to the animals, which were kept in controlled settings with a 12 h light/dark cycle, a temperature of 22 ± 2 °C, and a relative humidity of 50 ± 10%. The rats were given seven days to acclimate before treatment.

#### 2.11.1. Radiant Heat Tail-Flick Latency (TFL) Test

The in vivo anesthetic efficacy of the developed formulation was assessed in Wistar albino rats using the radiant heat TFL test, a validated method for evaluating nociceptive threshold and analgesic potential [33]. This method is widely employed for assessing the duration and intensity of analgesic or anesthetic activity in preclinical studies involving rodents, as supported by previous reports on LAs and pain models [34]. Animals were randomly assigned to three groups (n = 6 per group): Group I (Control): Received a subcutaneous injection of blank nanoemulgel. Group II (Test): Received the injectable LD-loaded nanoemulgel (12.6 mg/kg). Group III (Standard): Received a 2% LD HCl injection (12.6 mg/kg). Each formulation was administered subcutaneously at the distal 3 cm of the rat tail. Tail-flick latency was measured using a tail-flick analgesiometer (e.g., IITC Life Science Model 33, Woodland Hills, CA, USA) before administration (baseline) and at 30, 60, 90, 120, 150, 180, 210, and 240 min post-administration. A cut-off latency of 10 s was applied to prevent thermal injury to the tissue.

#### 2.11.2. Thermal Hind Paw Hyperalgesia Study

The analgesic efficacy of the nanoemulgel formulation was evaluated using a thermal paw withdrawal (Hargreaves) test, a well-established method for assessing thermal hyperalgesia in rodent models [35]. Baseline paw withdrawal latencies were recorded for both hind paws prior to treatment to ensure intra-subject control and enable accurate comparison of post-treatment responses. Following baseline assessment, each rat received a subplantar injection of the test formulation into one hind paw. Animals were then placed individually on a glass platform within a Plexiglas chamber and allowed a 5 min acclimatization period. A focused infrared radiant heat source (temperature range 50–52 °C) was applied to the plantar surface of the injected paw using a Hargreaves apparatus, and the withdrawal latency (in seconds) was recorded-defined as the time from onset of the thermal stimulus to paw withdrawal. To prevent tissue damage, a cutoff time of 20 s was enforced. Measurements were taken alternately from both hind paws, with a minimum 5 min inter-stimulus interval to prevent sensitization or desensitization. Each paw was tested five times per session, resulting in ten total observations per animal for each time point. Post-injection assessments were conducted at 30, 60, 90, 120, 150, 180, 210, and 240 min. The values are reported as mean ± standard deviation, based on five consecutive withdrawal latency determinations per paw. Prolongation of withdrawal latency in the treated paw, relative to baseline and control, was interpreted as indicator of analgesic efficacy.

#### 2.11.3. Cardiovascular Safety Studies

Baseline cardiovascular parameters, including heart rate, rhythm, and blood pressure, were recorded for all animals before administration of the formulations to enable accurate post-treatment comparisons [36,37]. For ECG monitoring (Kent Scientific, Torrington, CT, USA), a non-invasive tunnel system was employed to continuously record electrocardiograms. Electrodes were positioned on the skin at predetermined sites to capture heart activity, allowing real-time monitoring. The ECG data were analyzed for heart rate, rhythm, and potential arrhythmias or other abnormal cardiac events. For blood pressure monitoring (Emka Technologies, Paris, France), the non-invasive tail-cuff method was used to measure systolic, diastolic, and mean arterial pressure. Blood pressure was recorded through a software-based system to ensure accuracy and continuous data collection, with measurements taken at baseline and following treatment.

### 2.12. Data Analysis

Unless otherwise specified, the experiment data are the mean ± standard deviation of three studies. Statistical analysis was performed using repeated-measure analysis of variance (ANOVA) followed by one-way ANOVA, with *p* < 0.05 considered significant.

## 3. Results and Discussion

### 3.1. HPLC Analysis of Lidocaine

**Figure 2:**
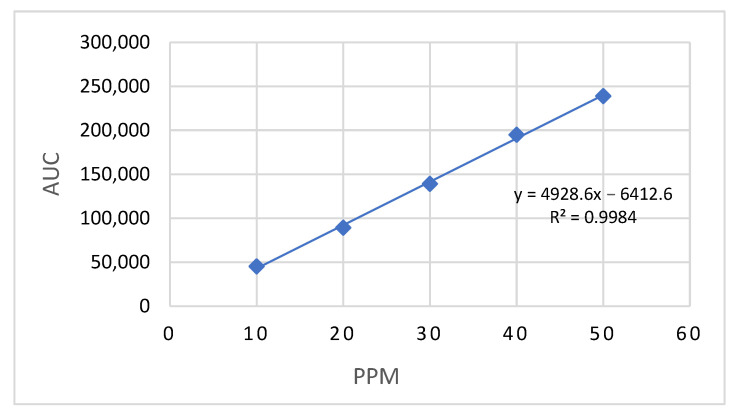
Calibration curve of LD-loaded nanoemulsion by HPLC at 273 nm.

A validated HPLC procedure was used to analyze LD. The LD standard solution had an average retention time of 6.2 min, while the formulated sample showed 6.5 min, both consistent with values reported in the literature [38]. A calibration curve (Figure 2) was constructed using concentrations ranging from 10 to 50 µg/mL, yielding a high linearity with a coefficient of determination (R^2^) of 0.9984 and a regression equation of y = 4928.6x − 6412.6. Method validation was carried out as per the recommendations of ICH Q2 [39], with the limit of quantification (LOQ) and limit of detection (LOD) established at 7.3 μg/mL and 2.41 μg/mL, respectively. The coefficient of variation across the tested concentration range ranged from 0.10% to 0.64%, and the percentage recovery of LD was found to be between 97.06% and 104.75%. Method validation confirmed acceptable intra-day and inter-day precision and accuracy, demonstrating the reliability and reproducibility of the analytical procedure.

### 3.2. Solubility Assessment and Selection of Excipients

Experimental solubility screening was performed to identify the most suitable excipients that maximize drug loading and ensure formulation stability, even though literature reports and regulatory acceptance served as supportive guidance for nanoemulsion development. LD base, a weakly basic LA with a log P of ~2.26, is moderately lipophilic and exhibits poor aqueous solubility, reported as 4.1 mg/mL at 30 °C [40]. This limited water solubility necessitates incorporation into an oil phase and surfactant system to achieve sufficient drug loading in a nanoemulsion suitable for parenteral delivery. Such a formulation not only enhances solubility and stability but also offers the potential to prolong drug action and reduce the frequency of administration. The successful development of an o/w nanoemulsion suitable for parenteral delivery requires careful selection of biocompatible excipients, particularly the oil phase, to maximize drug loading and ensure formulation safety and efficacy. To this end, the solubility of LD base was evaluated in various oils and surfactants to identify suitable excipients for nanoemulsion formulation. Among the oils tested, castor oil (538.0 ± 2.0 mg/mL), beeswax (347.0 ± 0.1 mg/mL), and triacetin (530.0 ± 0.7 mg/mL) demonstrated the highest solubility, followed by Miglyol^®^ 829 (221.0 ± 2.9 mg/mL), Miglyol^®^ 840 (210.5 ± 1.2 mg/mL), and Miglyol^®^ 812 N (171.5 ± 0.7 mg/mL). For surfactants, Tween 20 (186.1 ± 4.3 mg/mL), Tween 80 (161.0 ± 3.7 mg/mL), and soy lecithin (200.0 ± 3.4 mg/mL) showed moderate solubilization capacity.

Even though the solubility of Miglyol^®^ 812 N (Medium-chain triglycerides) is lower compared to other Miglyol^®^ series, it is the most widely accepted by the FDA and EMA for parenteral use. It is commonly used in injectable emulsions and approved products such as Diprivan^®^ due to its proven safety, biocompatibility, and regulatory compliance. In contrast, Miglyol^®^ 840 (propylene glycol diesters of caprylic/capric acid) lacks established regulatory approval for injectable applications, and Miglyol^®^ 829 (C8/C10 caprylic/capric triglycerides, high viscosity) is rarely utilized in parenteral formulations. Hence, Miglyol 812 N was chosen as the oil phase due to its biocompatibility and widespread use in parenteral formulations. Miglyol 812 is non-irritant, metabolically inert, and is categorized by the FDA as GRAS, making it suitable for intravenous and intramuscular routes [24]. Triacetin was avoided as the primary oil because of its partial water miscibility, which can promote drug partitioning into the aqueous phase and accelerate Ostwald ripening [41,42]. Castor oil was deprioritized because its high viscosity and ricinoleate content complicate emulsification and can increase the PDI [43]. Conversely, sufficient drug solubility in the chosen oil phase provides multiple formulation benefits, such as higher drug loading capacity, lower oil volume requirements, improved emulsion stability, and greater ease of sterilization and injectability [44]. Furthermore, optimizing the oil phase facilitates better control over droplet size and drug release kinetics, contributing to sustained anesthetic action upon parenteral administration.

Similarly, Tween 80, a hydrophilic non-ionic surfactant with a high hydrophilic–lipophilic balance (HLB ≈ 15), was selected for the nanoemulsion formulation owing to its adequate solubilizing capacity for LD base (~161 ± 3.1 mg/mL) and its well-established safety profile for parenteral use. Tween 80 is widely used in injectable emulsions and nanoemulsions because it effectively reduces interfacial tension, facilitates the formation of fine oil droplets, exhibits high solubilizing capacity, remains compatible with other surfactants and electrolytes, is stable across a wide pH range, and provides long-term stabilization of dispersed systems [45]. The effectiveness of Tween 80 in nanoemulsion-based drug delivery systems is well-supported in the literature. For example, an o/w parenteral emulsion containing metolazone (1 mg/mL), prepared by homogenizing soybean oil and L-lecithin with water using glycerin and Tween 80 as stabilizers and surfactant, respectively, exhibited a mean droplet size of 157.13 ± 1.52 nm and superior physical stability [46]. Glycerin is a pharmaceutically acceptable excipient in parenteral formulations, commonly employed in intravenous lipid emulsions, and was chosen over normal saline to avoid potential ionic destabilization of nanoemulsion droplets through compression of the electric double layer and disturbance of the interfacial film [47,48]. Tween 80 is preferred over Tween 20 for intravenous nanoemulsions because its oleate (C18:1) tail provides stronger interfacial affinity and oil solubilization with typical triglyceride oils, enabling smaller, more stable droplets at practical surfactant levels; this choice is further supported by extensive parenteral precedent in the literature [49]. Beeswax was excluded because, as a solid wax, it is unsuitable for clear parenteral o/w nanoemulsions and is prone to particulates/crystallinity upon cooling.

Soy lecithin (phosphatidylcholine-rich), with an HLB value of approximately 4–9, is widely used as a biocompatible emulsifier or co-surfactant in parenteral o/w emulsions, where it forms a stabilizing interfacial film around oil droplets and supports colloidal stability in intravenous nanoemulsion systems [50]. Lecithin-based emulsions have also demonstrated excellent injectability, low hemolytic activity (<5%), and stability under sterilization and freeze–thaw conditions [51]. Thus, the rational selection of oil and emulsifying agents was primarily guided by both the solubility profile of the LD base and the stringent safety requirements for parenteral dosage forms. The surfactant system, comprising Tween 80 (polysorbate 80) and soy lecithin, both accepted for parenteral use, forms a robust mixed interfacial film and reliably lowers interfacial tension [52,53].

The choice of surfactant based on its HLB is critical for stabilizing nanoemulsions. The HLB value of a surfactant determines whether it promotes o/w or water-in-oil emulsions, regardless of the dispersed phase concentration [54]. While surfactants do not make emulsions thermodynamically stable, they enhance kinetic stability by preventing phase separation through mechanisms like creaming, flocculation, and coalescence. For o/w systems, surfactants with HLB values above 8 are preferred, as they lower interfacial tension and improve thermodynamic stability [54,55]. An ideal HLB of 9 was obtained by combining soy lecithin (HLB 7) and Tween 80 (HLB 15) in a 3:1 Smix ratio. According to reports, Ropinirole oil-in-water nanoemulsions are developed and stabilized more easily when a low-HLB surfactant, like carbitol, is combined with a high-HLB surfactant, like Tween 80 [54].

In summary, the moderate lipophilicity of LD base (log P ~2.26) necessitates careful excipient selection, as it is insufficiently soluble in water yet not highly lipophilic. Medium-chain triglycerides (Miglyol^®^ 812 N) provide a suitable lipophilic environment for solubilization, while a mixed surfactant system of Tween 80 and lecithin reduces interfacial tension and stabilizes the oil droplets. This combination corresponds with the drug’s log P profile, ensuring efficient drug loading, droplet stability, and suitability for parenteral administration.

### 3.3. Development of LD-Loaded Nanoemulsion

Minimizing the formulation volume is essential for parenteral dosage forms because of the strict volume limitations associated with injections. Hence, achieving a high drug payload capacity is critical to ensure that the therapeutic dose can be effectively delivered within the limited permissible volume. The capacity to encapsulate and maintain the drug in a solubilized state depends largely on its solubility in individual formulation components, particularly the oil phase. Hence, optimizing the oil selection and concentration was done to enhance drug solubilization and reduce the overall formulation bulk, thereby enabling delivery of therapeutic doses within the restricted injectable volume. Notably, relying heavily on surfactants or co-surfactants for solubilization can lead to drug precipitation upon dilution, particularly under physiological conditions such as in the gastrointestinal tract or local tissues, due to reduced solvent capacity after dilution. LD-loaded nanoemulsions (S1–S3) were developed by systematically varying the oil phase concentration, 7.5% in S1, 8% in S2, and 8.5% in S3, while maintaining a constant 4% Smix (Tween 80 and soy lecithin in a 1:3 ratio). The water content was adjusted accordingly to complete the formulation to 100%, allowing the evaluation of the oil phase’s impact on drug solubilization, physicochemical behavior, and stability of the nanoemulsions, while keeping surfactant levels constant.

During the preliminary phase of formulation development, the concentration of the oil phase was varied in narrow increments. This was based on pilot trials showing that concentrations below 7.5% and above 8.5% consistently produced unstable dispersions with droplet sizes exceeding 1 μm, which did not qualify as nanoemulsions. Consequently, the focus of the present study was not to establish the absolute optimum oil concentration but rather to identify the most effective ratio between oil and surfactant, in combination with the aqueous phase, to generate a stable nanoemulsion system. During preliminary studies, probe sonication was carried out at 30% amplitude with a pulse rate of 25 while varying the sonication time (2–10 min) and frequency. The results indicated that a 5 min treatment consistently yielded nanoemulsions with the smallest droplet size and an acceptable PDI, confirming a uniform droplet distribution. Extending the sonication period beyond 5 min did not reduce the size of droplets and increased the possibility of thermal breakdown, whereas low durations led to inadequate emulsification. This rationale supports the selected sonication parameters, as they ensured efficient processing, droplet size reduction, and PDI optimization. The influence of sonication parameters, including frequency and duration, on nanoemulsion characteristics has been well documented in previous studies [56,57]. Pre-treatment with a high-shear homogenizer provided initial homogenization, which enhanced the effectiveness of subsequent ultrasonication in reducing droplet size.

### 3.4. Characterization of Nanoemulsion

Evaluating the pharmaceutical properties of nanoemulsions is vital for confirming their stability, effectiveness, and potential for pharmaceutical use. The physicochemical characterization of nanoemulsions (S1–S3) was carried out, and the results are presented in Table 1. In nanoemulsion formulations, drug content (%) indicates uniform distribution, whereas pH plays a critical role in determining stability and biocompatibility. Drug content was calculated relative to the theoretical drug amount incorporated into the formulation, with 100% corresponding to the nominal drug content based on the composition. The formulations exhibited drug contents ranging from 96% to 98%, indicating nearly complete incorporation of the drug into the nanoemulsion, and a pH of approximately 5.6, which is compatible with subcutaneous administration and suggests a low likelihood of local tissue irritation [58]. Transmittance serves as an indicator of nanoemulsion clarity, whereas dilution capability evaluates formulation stability upon aqueous dilution. The size of the droplets (nm) and PDI are critical determinants of the release of API, bioavailability, and stability. *ζ* potential (mV) provides information on electrostatic stabilization, thereby preventing droplet aggregation. Viscosity (cP) influences rheological behavior, directly affecting flow properties and ease of administration [10]. High transmittance values (>95%) confirmed the optical clarity of the formulations and indicated the presence of nanometer-sized droplets (206–293.73 nm) with homogeneous dispersion of the oil phase, allowing efficient light transmission despite the relatively broad PDI values (0.4–0.5). It was acknowledged that the relatively higher PDI values indicate moderate polydispersity; hence, further studies should focus on optimizing formulation parameters to achieve a narrow size distribution for improved reproducibility and optical clarity. The *ζ* potential of the formulation ranged from –60 mV to –70 mV, indicating that a pronounced electrostatic repulsion between droplets plays a key role in stabilizing nanoemulsion by preventing aggregation and coalescence. Droplet stabilization is achieved by non-ionic surfactants such as Tween 80, which form a hydrophilic shell that provides steric hindrance and minimizes droplet coalescence [59]. However, the significant negative ζ potential is attributed to the presence of soy lecithin, which contains negatively charged phospholipid head groups as reported elsewhere [60]. Thus, the overall stability of the system is supported by a combination of electrostatic and steric stabilization mechanisms. Mixed-surfactant nanoemulsions exhibit enhanced stability against environmental fluctuations due to dominant repulsive forces over attractive ones (hydrophobic and van der Waals forces). The measured viscosity of the prepared nanoemulsion ranged from 33 to 38 cP, which falls within the acceptable range for parenteral administration, ensuring ease of injection and minimal resistance during delivery. The prepared nanoemulsion’s distinct structural and physicochemical characteristics were demonstrated by the results. Studies have shown that kinetically stable nanoemulsions with droplet sizes below 500 nm serve as advanced drug delivery systems, improving solubility, bioavailability, and targeted therapeutic delivery [61].

**Figure 3:**
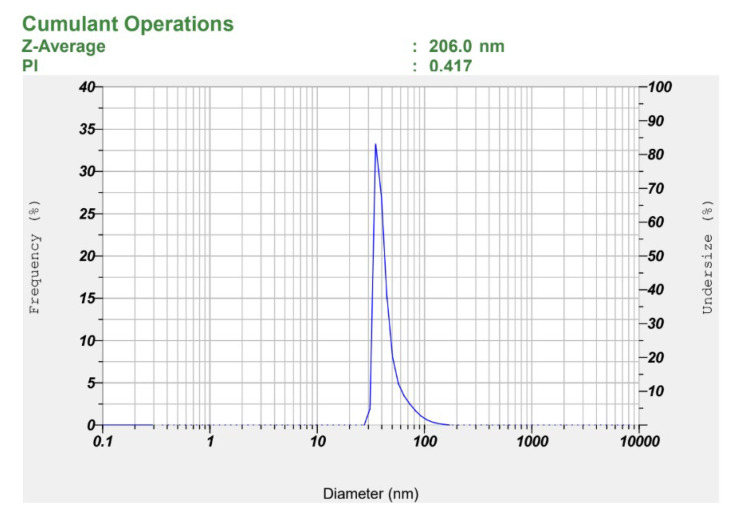
Observed particle size of the selected LD-loaded nanoemulsion (S1).

A parallel comparison of S1–S3 nanoemulsions formulated with identical Smix (4% Tween-80:soy lecithin, 1:3) and increasing oil (7.5%→8.0%→8.5%) shows that S1 provides the most favorable developability profile: it has the smallest droplet size (206 ± 7.5 nm) and lowest PDI (0.40 ± 0.04) versus S2 (294 ± 6 nm; 0.51 ± 0.03) and S3 (227 ± 4 nm; 0.49 ± 0.01) (Figures S1 and S2), consistent with the quality expectation that smaller size and lower PDI improve stability, drug solubilization, and performance in lipid nanocarriers [9,62]. It is worthwhile to note that minimizing droplet size in injectable nanoemulsions is particularly important, as smaller droplets can reduce local tissue distension and inflammatory responses that contribute to injection site pain, a key factor in enhancing patient comfort and adherence [63]. As illustrated in Figure 3, the globule size distribution is both narrow and unimodal. Nanoemulsions with uniform and small droplet sizes are highly desirable for intravenous or subcutaneous administration due to their superior physicochemical and biopharmaceutical properties. A small and narrowly distributed droplet size (typically < 200 nm) ensures better physical stability; reduces the risk of creaming, coalescence, or phase separation; and enables smooth injectability through fine-gauge needles. For instance, parenteral nanoemulsions such as *Diprivan^®^* (propofol injection) utilize lipid droplets within the 150–200 nm range to achieve rapid onset of action with minimal injection site irritation [64]. Furthermore, a narrow distribution of particle sizes reduces the possibility of droplet coalescence and Ostwald ripening, while improving the stability of nanoemulsions [41].

**Figure 4:**
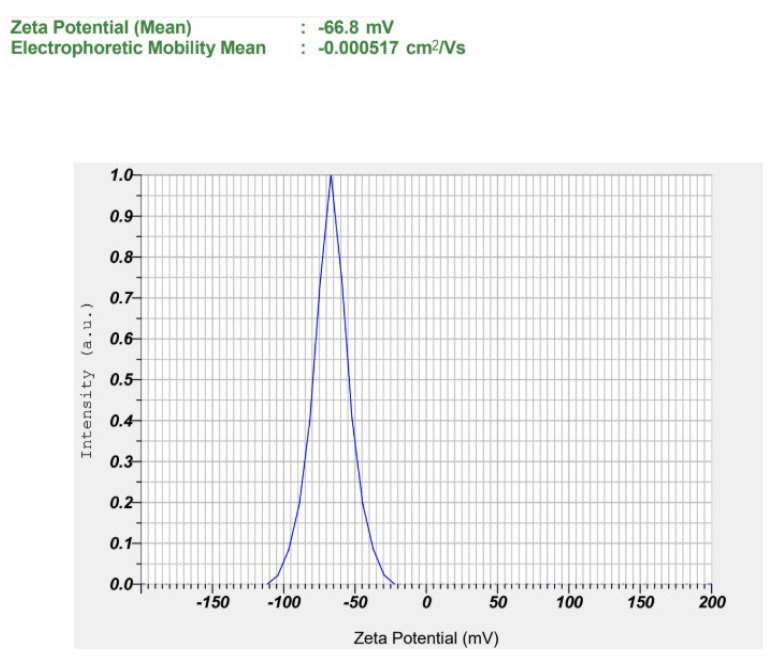
Observed zeta potential of the selected LD-loaded nanoemulsion (S1).

Furthermore, S1 showed a highly negative ζ potential (−66.67 ± 0.42 mV), suggestive of strong electrostatic repulsion between droplets, which contributes to enhanced physical stability over time by preventing droplet aggregation, creaming, and coalescence [65]. The relatively higher water content in S1 (84.25%) compared to S2 (83.75%) and S3 (83.25%) may have contributed to enhanced dispersion of the oil phase and improved solubilization of the surfactant mixture, thereby decreasing the likelihood of droplet aggregation or coalescence under stress stability conditions. Figure 4 presents representative ζ potential images of the selected formulation. ζ potential images of S2 and S3 are presented in Figures S3 and S4. In addition, S1 also had the lowest viscosity (33.1 cP vs. 36.9–38.0 cP for S2–S3), predicting lower injection force at a fixed needle gauge and flow rate [66]. Moreover, S1 showed high transmittance (96.8%), drug content (97.6%), EE (98.91%), DL (9.32%), with suitable pH (~5.8) and >20-fold dilution stability, consistent with a transparent o/w nanoemulsion. Because Smix was held constant, the observed trend (larger droplets and higher viscosity) with increasing oil reflects reduced interfacial coverage per unit oil at higher dispersed-phase fractions [62]. Collectively, the superior aqueous content, enhanced dispersion, improved surfactant solubilization, stability under stress, and favorable physicochemical parameters establish S1 as the lead formulation for gel incorporation and in vivo evaluation. Although various physicochemical parameters were evaluated, stability under physiological conditions, after dilution, and during extended storage was not assessed in the present study. These aspects are recognized as important for clinical translation and will be systematically investigated in future studies.

### 3.5. Kinetic Stability Studies

Nanoemulsion physical stability is assessed using a comprehensive accelerated stress protocol, comprising repeated freeze–thaw cycles, heating–cooling cycles, and centrifugation, routinely applied to emulate worst case excursions and mechanical stresses that may occur during transport and storage. These orthogonal challenges probe distinct failure modes: ice crystal-induced coalescence and phase separation (freeze–thaw), thermally driven Ostwald ripening and viscosity shifts (heating cooling), and creaming/sedimentation under forced gravity (centrifugation). Formulations were identified with a check mark (Table 2) if they exhibited no signs of instability during the respective stress tests. Stability was confirmed when centrifugation (3500 rpm for 30 min) produced no phase separation, creaming, or sedimentation; heating–cooling cycles (6 cycles between 4 °C and 45 °C, 48 h each) caused no cracking, phase separation, or visible changes; and freeze–thaw cycles (3 cycles between –21 °C and 25 °C, 24 h each) resulted in no precipitation, turbidity, or phase instability. All nanoemulsion formulations (S1–S3) remained stable, with no detectable signs of instability observed under stress conditions. The absence of nanoscale aggregation and macroscopic phase separation indicates that the excipients and interfacial surfactant film remained intact, with no crystallization or other phase transitions under thermal and mechanical stresses. These findings align with literature reports emphasizing the importance of interfacial surfactant behavior and crystallization resistance in ensuring emulsion stability under thermal stress [67]. The binary surfactant mixture plays a crucial role in enhancing emulsion stability through the rapid formation of a robust and cohesive interfacial film at the oil–water interface. This effect is particularly evident during storage and under high shear conditions, where the tightly packed interfacial layer provides mechanical strength and improved steric stabilization [68].

Previous studies have shown that nanoemulsions with an ideal balance of aqueous and surfactant/oil components generate a strong interfacial coating around particles, increasing their resilience to temperature and mechanical stress. In this context, the superior freeze–thaw stability of formulation S1 can be attributed to its ideal composition of oil, Smix, as well as water, which promotes the formation of a resistant interfacial layer and decreases phase separation. These observations align with previous reports highlighting the role of composition in the thermal stability of nanoemulsions [69,70]. In addition, droplet size and PDI were re-evaluated after the stress tests, showing no significant deviation (*p* > 0.05), thereby validating that the nanoemulsions retained their colloidal stability under stress conditions. Overall, S1 was selected as the suitable LD-loaded nanoemulsion due to its favorable combination of small droplet size, uniform size distribution, high drug content, suitable pH, excellent transmittance, and stable surface charge, along with good dilution potential and lower viscosity, making it the most stable and promising formulation compared to S2 and S3.

### 3.6. TEM

**Figure 5:**
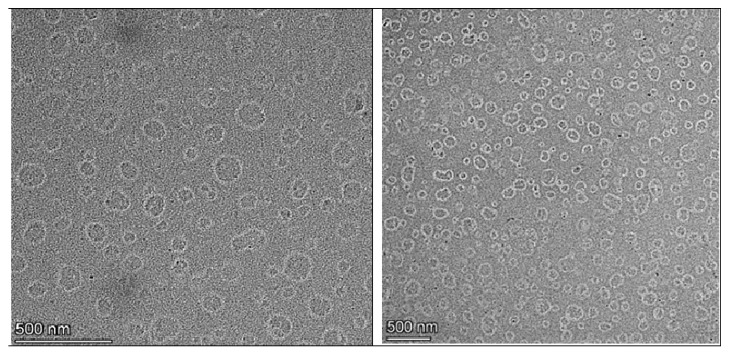
TEM image of LD-loaded nanoemulsion (S1).

Morphological investigations using TEM give visible validation of the nano-scale dimensions and structural integrity of the developed nanoemulsion formulation. The TEM image of the LD-loaded nanoemulsion (Figure 5) clearly illustrates the presence of uniformly distributed spherical droplets with well-defined boundaries. The droplets appear as dark, electron-dense structures against a bright background, consistent with the negative staining technique commonly employed in TEM analysis of lipid-based nanocarriers. The scale bar in the image corresponds to 500 nm, and the average droplet diameter was visually estimated to be approximately 200–210 nm. These values align closely with the results obtained from dynamic light scattering, which reported a hydrodynamic diameter of 206 ± 7.50 nm prior to freeze–thaw cycling and 210 ± 8.50 nm post-thaw. The minor discrepancy between the two methods is expected and attributed to the differing measurement principles: dynamic light scattering measures the hydrodynamic diameter, including the solvation layer around the particles, whereas TEM captures the actual physical diameter of the dried droplets under vacuum conditions. Notably, the nanoemulsion droplets were dispersed homogeneously throughout the field with no signs of aggregation, coalescence, or irregular morphology, suggesting that the formulation is physically stable with effective steric and electrostatic stabilization provided by surfactants like Tween 80 and soy lecithin. These findings are consistent with previously published reports, which describe nanoemulsion droplets as monodispersed, spherical vesicles typically ranging between 100–300 nm when visualized by TEM [71].

### 3.7. Characterization of Nanoemulgel

To achieve prolonged anesthetic action and controlled drug release, the selected LD-loaded nanoemulsion was incorporated into a thermosensitive in situ gel system. The gel matrix was formulated using Poloxamer 407, a nonionic, synthetic triblock copolymer of PEO and PPO widely employed for its thermoreversible gelling behavior, low toxicity, and biocompatibility in parenteral formulations [72].

#### 3.7.1. pH and Drug Content

The selected nanoemulgel formulation’s pH is 6.02 ± 0.13, which is well within the acceptable range for parenteral use, indicating good potential for local tolerability and minimal risk of tissue irritation at the injection site [24]. As the formulation exhibited a pH close to physiological levels, it is expected to cause minimal tissue irritation and thereby enhance patient compliance. In addition, the high drug content (97.61 ± 1.48%) reflects efficient drug loading.

#### 3.7.2. Viscosity

A thixotropic viscosity profile is essential for injectable depot formulations to ensure adequate flow through a syringe during administration and to enable rapid gelation at the injection site, thereby supporting sustained anesthetic release. In thermoresponsive systems based on Poloxamer 407, viscosity is a key parameter governing the sol-to-gel transition at physiological temperatures [73,74]. The viscosity of the LD-loaded nanoemulgel was evaluated at 37 °C across a range of controlled shear rates (50–250 rpm) to mimic physiological conditions. The formulation exhibited a distinct shear-thinning behavior, characterized by a progressive decrease in viscosity with increasing shear rate. Such pseudoplastic flow is highly desirable for injectable systems, as it ensures low resistance during administration through a syringe needle while maintaining the structural integrity of the gel depot at the target site after injection. Under a fixed shear rate of 50 rpm, the viscosity was found to be 17,184.00 ± 103.00 cP, confirming the formation of a consistent gel matrix with adequate mechanical strength under physiological temperature. This rheological profile suggests that the LD-loaded nanoemulgel is capable of providing both ease of injection and sustained in situ retention, which are critical for achieving prolonged therapeutic activity.

#### 3.7.3. Sol–Gel Transition and Injectability Testing

Poloxamer 407 is the only FDA-approved inactive ingredient guide polymer for parenteral applications that demonstrates a concentration-dependent sol–gel transition. At low concentrations (10–12%), gelation occurred at higher temperatures (50–60 °C) with slow gelation times (~15 min) (Table 3). Increasing the concentration (14–20%) progressively lowered the gelation temperature, reaching physiologically relevant levels (35–37 °C) at 18–20%, with rapid gelation (less than 5 min). This behavior can be attributed to enhanced micellar packing and PPO dehydration. Upon heating or increasing concentration, hydrophobic PPO blocks dehydrate and aggregate to form micellar cores, while hydrophilic PEO blocks remain hydrated as coronas. These micelles then arrange into cubic or hexagonal phases, yielding hydrogels that can revert to sol upon cooling [75]. Earlier research has explored poloxamer based hydrogels as carriers for the in situ delivery of LAs [76]. Lower Poloxamer concentrations (10–16%) exhibited delayed gelation and weaker gel strength. Although the 20% formulation gelled more rapidly (~3 min), this occurred near room temperature (25 ± 0.5 ^°^C), increasing the risk of premature gelation during handling and storage. In contrast, 18% Poloxamer 407 remained in sol form at room temperature but underwent a rapid sol–gel transition (≤5 min) at physiological temperature (~37 °C). The gelation time of thermo-sensitive hydrogels, a key factor for clinical applicability, is primarily influenced by the concentration and composition of the formulation materials [77]. This concentration offered an optimal balance of gelation kinetics, mechanical strength, injectability, and controlled drug release. As reported elsewhere, LD release can be tailored by adjusting the concentration of Poloxamer 407, with the gel system prolonging residence time, sustaining release, and enhancing therapeutic efficacy [76]. Accordingly, 18% Poloxamer 407 was selected as the most pharmaceutically suitable formulation for fabricating in situ gel of LD-loaded nanoemulsion (S1) for subsequent in vivo studies. The nanoemulgel obtained was smooth, homogeneous, and translucent, showing no visible phase separation or precipitation throughout the study, thereby demonstrating physical stability and formulation uniformity. Literature evidence suggests that this concentration provides the ideal balance for rapid sol–gel conversion at physiological temperature (~37 °C, <1 min) alongside favorable injectability and syringeability [78,79].

To ensure a pharmaceutically acceptable injectable formulation, the injectability, referring to the ease of syringe ejection through a needle, must be assessed. Injectability is a key parameter, particularly for high-viscosity formulations. The injectability of the nanoemulgel formulation was assessed using a Brookfield CT3 Texture Analyzer with 23 G and 26 G syringe needle systems. The average force required for administration was 2.45 ± 0.04 N with the 23G needle and 3.60 ± 0.18 N with the 26 G needle, obtained from the graph when plotted against the injection speed of 100 mm/min. Forces obtained in the range of 0–10 N indicate very easy injectability and suggest that further reduction in needle size is feasible. Accordingly, the nanoemulgel formulation demonstrated very easy injectability, with both needle gauges requiring minimal force for extrusion.

### 3.8. In Vitro Drug Release

**Figure 6:**
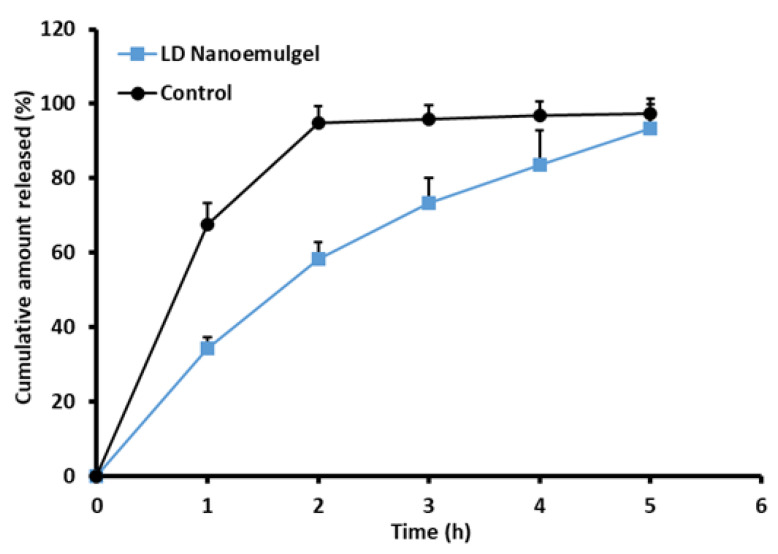
Release of drug from prepared LD-loaded nanoemulgel vs. the standard 2% LD-HCl (n = 3).

Drug release from the nanoemulgel plays a vital role in sustaining therapeutic efficacy, maintaining adequate drug levels at the target site, and improving patient compliance. Controlled release from the nanoemulgel matrix contributes to prolonged residence time, reduced dosing frequency, and lower systemic side effects. Figure 6 depicts the cumulative release profile of LD over different time intervals, comparing the nanoemulgel formulation with the standard 2% LD-HCl injection. The in vitro release study revealed that LD release from the formulated nanoemulgel reached 93.41 ± 6.34% at the end of 5 h, whereas the standard 2% LD-HCl injection exhibited a comparatively higher release of 94.76 ± 4.56% up to 2 h. This difference in release rate suggests that the nanoemulgel formulation effectively modulates drug diffusion, offering a more sustained release profile relative to the conventional injectable solution. On the other hand, the drug (LD) is dissolved in the oil phase; hence, it exists in a solubilized state inside the developed nanoemulsion droplets, which are further dispersed within the hydrophilic three-dimensional poloxamer gel matrix. Such systems can provide steady drug release behavior, as drug release is governed initially by partitioning and diffusion from the nanoemulsion droplets, followed by sustained diffusion through the hydrated poloxamer network. In addition, hydrogen bond interactions between the drug and the polymer matrix play a key role in sustaining the prolonged release profile of the formulation [80]. Moreover, these observations are consistent with earlier reports in the literature demonstrating prolonged anesthetic action and improved local drug retention using nanoemulgel-based delivery systems [76,81]. Release kinetics analysis indicated that the drug release from the formulation followed the Higuchi model with a high r^2^ value (0.992). This best fit indicates that the release of LD from the nanoemulgel formulation is primarily governed by Fickian diffusion through the hydrated polymeric network. Overall, the synergistic effect of nanoemulsion encapsulation and gel-matrix diffusion control provides a mechanistic basis for the sustained release profile of LD, supporting its potential to enhance therapeutic outcomes and patient compliance. Although the nanoemulgel was evaluated against the standard LD solution as a clinically relevant reference, additional studies with the nanoemulsion alone are required to fully clarify the role of the gel matrix in modulating drug release.

### 3.9. Radiant Heat Tail-Flick Latency Test

**Figure 7:**
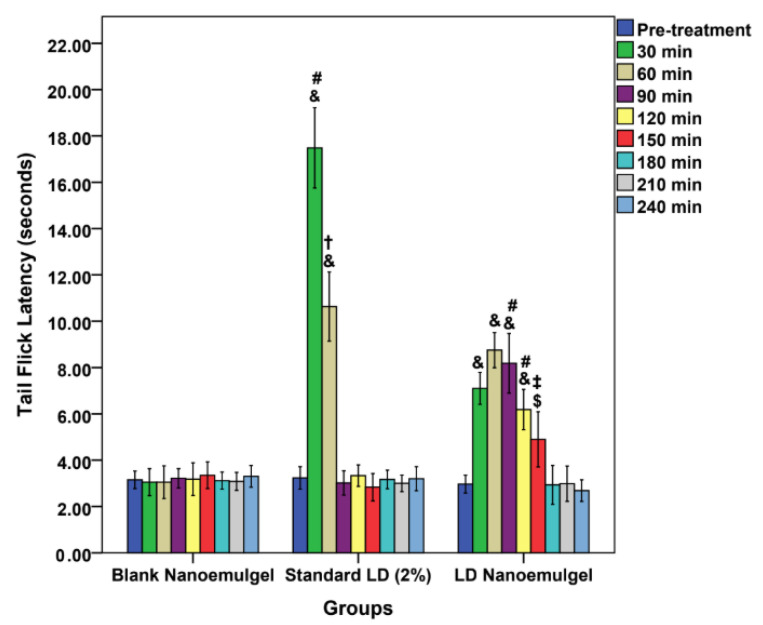
Tail flick latency vs. time passed; comparison of different formulations: Blank nanoemulgel (negative control), standard LD 2% (positive control), and LD nanoemulgel (test group); & *p* < 0.001, $ *p* < 0.05, one-way ANOVA, Tukey’s HSD, compared with blank nanoemulgel group; # *p* < 0.001, ‡ *p* < 0.01, † *p* < 0.05, one-way ANOVA, Tukey’s HSD, comparing standard LD 2% and LD nanoemulgel groups.

In the present study, nociceptive assays, namely the Hargreaves paw withdrawal and TFL tests, were employed to assess the sustained release characteristics of parenterally administered LD-loaded nanoemulgel. While traditionally considered analgesic models, these assays directly reflect the primary pharmacological action of LAs, which is the blockade of voltage gated sodium channels in nociceptive Aδ and C fibers. This action results in the loss of pain and thermal sensation prior to broader sensory or motor effects. Evidence of prolonged analgesia in these validated models is widely regarded as a reliable surrogate for anesthetic efficacy, consistent with prior findings [82]. TFL test primarily evaluates spinal reflex nociception and centrally mediated analgesic effects, providing complementary evidence to the Hargreaves paw withdrawal test [33,83]. In the present study, the blank nanoemulgel group (control) maintained latency values close to baseline throughout the 240 min observation period (~3.0–3.3 s), again confirming the absence of analgesic action (Figure 7). The LD injection (standard) produced a sharp peak response at 30 min (17.48 ± 1.73 s, *p* < 0.001 vs. control), indicating rapid analgesic onset. However, this effect diminished significantly by 60 min (10.63 ± 1.49 s) and returned nearly to baseline at 90 min (3.02 ± 0.52 s), consistent with the short duration of action reported for LD in vivo [84]. In contrast, the LD-loaded nanoemulgel (test) demonstrated a delayed but markedly prolonged analgesic response. Latency values increased moderately at 30 min (7.1 ± 0.69 s, *p* < 0.001 vs. control), remained elevated at 60 min (8.75 ± 0.76 s), and were still significantly higher than both negative and positive controls at 90 min (8.18 ± 1.29 s). Importantly, appreciable analgesic activity persisted up to 150 min (4.9 ± 1.19 s), after which latency values gradually returned toward baseline by 180–240 min. This extended duration of analgesia suggests that the nanoemulgel system facilitates a sustained release of LD, likely due to the dual role of the lipid phase in drug solubilization and the gel matrix in controlling diffusion kinetics. Such sustained activity in TFL is in agreement with prior literature demonstrating that nanoemulsion-based carriers enhance percutaneous absorption and prolong the pharmacodynamic effects of anesthetics through depot-like release behavior [85,86,87].

### 3.10. Thermal Hind Paw Hyperalgesia Study

**Figure 8:**
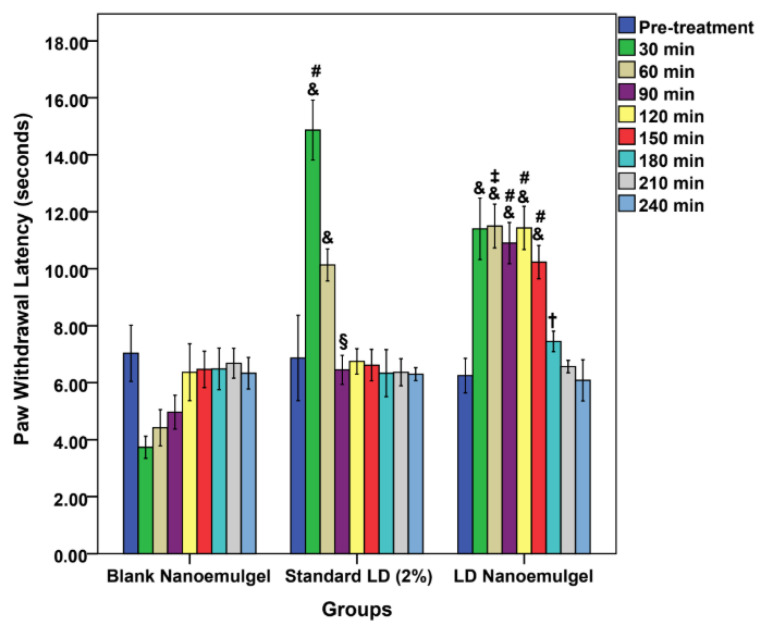
Paw withdrawal latency vs. time passed; comparison of different formulations: Blank nanoemulgel (negative control), standard LD 2% (positive control), and LD nanoemulgel (test group); & *p* < 0.001, § *p* < 0.01, one-way ANOVA, Tukey’s HSD, compared with blank nanoemulgel group; # *p* < 0.001, ‡ *p* < 0.01, † *p* < 0.05, one-way ANOVA, Tukey’s HSD, comparing standard LD 2% and LD nanoemulgel groups.

The paw withdrawal latency test revealed significant differences in the onset, intensity, and duration of analgesic activity among the groups. At baseline (0 min), all groups displayed comparable latencies (7.03 ± 0.99 s for control, 6.87 ± 1.5 s for standard, and 6.25 ± 0.61 s for test), with no statistical differences (control vs. standard: *p* = 0.962; control vs. test: *p* = 0.449; standard vs. test: *p* = 0.602) (Figure 8). Following administration, a clear divergence in analgesic response was observed at different time points. At 30 min, the standard LD injection showed a pronounced increase in latency (14.87 ± 1.05 s, *p* < 0.001 vs. control), while the test group also exhibited a significant rise (11.4 ± 1.08 s, *p* < 0.001 vs. control). Notably, both treatments differed significantly (*p* < 0.001), with the standard producing a stronger but sharper peak. At 60 min, the standard latency decreased to 10.13 ± 0.56 s, whereas the test group maintained higher latency (11.5 ± 0.77 s, *p* = 0.007 vs. standard, indicating more sustained analgesia. By 90 min, the test group continued to show superior latency (10.9 ± 0.72 s, *p* < 0.001 vs. standard and control), whereas the standard dropped to 6.45 ± 0.51 s, nearing baseline. Importantly, the test formulation maintained significantly elevated latencies up to 150 min (11.43 ± 0.76 s at 120 min and 10.23 ± 0.59 s at 150 min, both *p* < 0.001 vs. control and standard). Even at 180 min, the test group retained a modestly higher latency (7.45 ± 0.36 s, *p* = 0.029 vs. standard), before gradually converging with controls at 210–240 min (no statistical differences). While the Blank Nanoemulgel group (negative control) initially exhibited a transient reduction in latency, likely due to injection prick pain and the exertion of full body weight onto the paws (3.73 ± 0.39 s at 30 min; 4.42 ± 0.64 s at 60 min; 4.97 ± 0.59 s at 90 min), the values gradually recovered to baseline by 120 min (6.37 ± 1 s) and remained stable thereafter. This finding confirms that the blank formulation itself did not exert any analgesic effect, thereby reinforcing that the prolonged increase in latency observed in the test group was attributable to the pharmacological action of LD released from the nanoemulgel matrix rather than the vehicle. These findings demonstrate that while LD injection exerts a rapid and intense effect, its action wanes within 60–90 min, consistent with its known short duration of activity [88]. In contrast, the LD nanoemulgel provides a more prolonged analgesic profile, sustaining significant activity up to 150 min, with residual effects observed at 180 min. The prolonged efficacy may be attributed to the nanoemulgel matrix that enables sustained drug release, enhanced permeation, and reduced clearance, as documented in previous reports on lipid-based nanoformulations for LAs [89]. Thus, the paw withdrawal latency and TFL outcomes strongly support the potential of LD nanoemulgel as a superior alternative to conventional injection for conditions requiring extended local analgesia.

### 3.11. Cardiovascular Safety Studies

**Figure 9:**
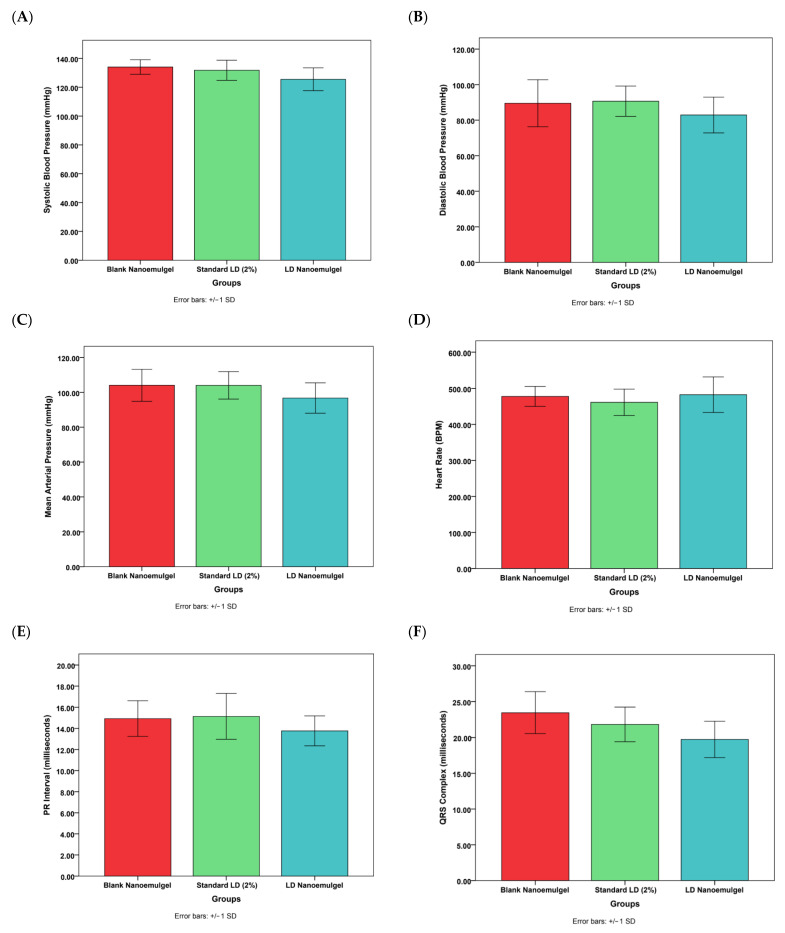

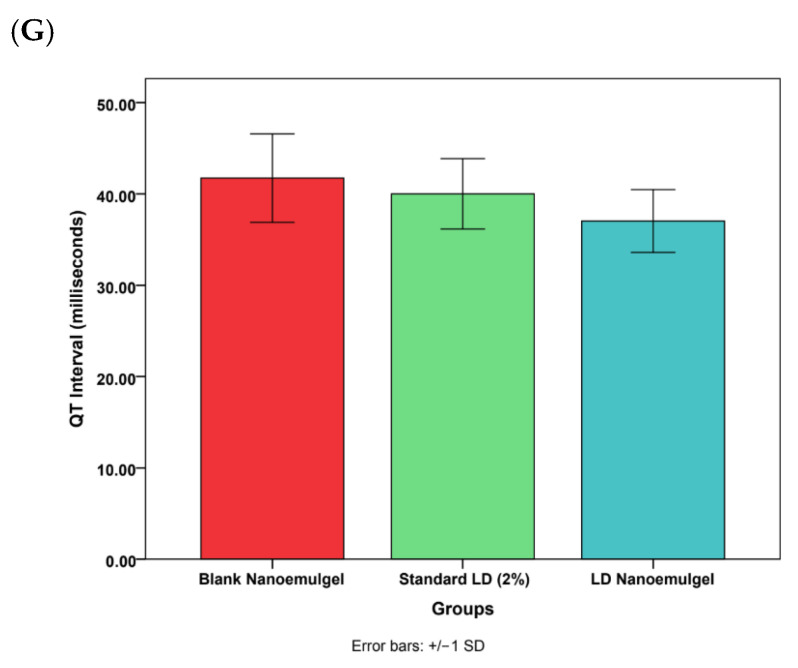
Various cardiovascular safety studies of blank nanoemulgel (negative control), standard LD 2% (positive control), and LD nanoemulgel (test group). (**A**) Systolic blood pressure, (**B**) Diastolic blood pressure, (**C**) Mean arterial pressure, (**D**) Heart rate, (**E**) PR interval, (**F**) QRS complex, (**G**) QT interval. Statistically insignificant from other formulations at *p* < 0.05.

Cardiovascular safety assessment showed no statistically significant differences between pre- and post-treatment values for blood pressure, ECG parameters, or heart rate, confirming that administration of LD nanoemulgel did not induce adverse cardiovascular effects (Figure 9). Representative dynamic ECG baseline waveforms and post treatment waveforms (Figures S5 and S6) along with corresponding quantitative statistical analysis of heart rate, PR interval, QRS duration, and QTc interval are presented in Table S1. Further, the developed LD nanoemulgel (test group) demonstrated clear therapeutic superiority over the marketed preparation, with a faster onset, prolonged duration of analgesia, and sustained nociceptive suppression, all achieved without significant cardiovascular alterations. These findings strongly support the translational relevance of the formulation for effective management of acute and procedural pain conditions.

While the present study primarily utilized thermal nociceptive models (Har-greaves and Tail-Flick), which are clinically relevant to inflammatory and postoperative pain, the absence of mechanical nociceptive assessment represents a minor methodological constraint rather than a limitation to interpretation. Furthermore, although extended ECG monitoring and serum drug concentration profiling beyond 24 h were not conducted, the available cardiovascular and functional data provide robust evidence of short-term safety and pharmacological efficacy. Future studies will expand on these parameters to further enhance mechanistic understanding and confirm long-term safety without detracting from the strength of the current therapeutic findings.

### 3.12. Stability Studies

The nanoemulsion (S1) as well as the corresponding nanoemulgel demonstrated stability during the three-month study period, underscoring the prospects for longer storage and clinical application. The S1 formulation consistently maintained clarity, with no evidence of turbidity or precipitation. In addition, no signs of instability, including phase separation, pH variation, viscosity changes, or fluctuations in drug content, were detected, confirming its robustness for pharmaceutical development. Similarly, the nanoemulgel remained stable for the duration of the study, showing no measurable alterations in any of the assessed parameters. Collectively, these findings underscore the feasibility of both formulations for parenteral drug delivery, ensuring reproducible therapeutic efficacy and safety during storage and use.

## 4. Conclusions

This study successfully designed and characterized an injectable LD nanoemulsion embedded in a thermoreversible poloxamer gel depot aimed at prolonging local anesthesia during dental and operative procedures. The selected formulation (S1) exhibited favorable physicochemical properties, including nanosized droplets (~206 nm) with a uniform distribution and a high ζ potential (−66.67 mV), with no evidence of drug–excipient interactions. Incorporation into the thermoreversible gel system yielded a smooth, homogeneous depot formulation with suitable injectability. In vitro release confirmed sustained LD release over 5 h (vs. 2 h for standard LD-HCl injection), while in vivo tail-flick and paw withdrawal tests showed prolonged analgesic effects lasting up to 150 min (vs. 90 min for standard LD). Cardiovascular safety assessments revealed no significant alterations in ECG or blood pressure parameters, supporting the in vivo safety of the developed formulation. These findings validate the working hypothesis that embedding an LD nanoemulsion in a gel depot can extend the duration of analgesia while maintaining cardiovascular safety. Overall, the injectable in situ nanoemulgel provides a promising, patient-friendly alternative for prolonged anesthetic delivery in dental and operative settings, reducing the need for repeated injections and enhancing procedural comfort. A limitation of this study is the challenge of sterilization stability in thermoresponsive nanoemulgel systems. While aseptic preparation was used, subsequent studies will focus on developing optimized sterilization strategies to ensure both sterility and physicochemical stability. Although the present animal study focused on the optimized nanoemulgel as the intended parenteral dosage form, future investigations should include comparative evaluations with the conventional nanoemulsion to better delineate the specific contribution of the gel matrix to sustained release and prolonged analgesic efficacy.

## Figures and Tables

**Table 1:** Formulation composition and physicochemical characterization of LD-loaded nanoemulsions (S1–S3).

Component	S1	S2	S3
Oil phase (%)	7.5	8.0	8.5
Surfactant (%)	1.0	1.0	1.0
Co-surfactant (%)	3.0	3.0	3.0
Stabilizer (%)	2.25	2.25	2.25
Aqueous phase (%)	qs to 100	qs to 100	qs to 100
**Evaluation Parameters**			
Drug content (%)	97.61 ± 1.48	96.90 ± 1.13	96.04 ± 2.97
Entrapment efficiency (%)	98.91 ± 0.48	98.90 ± 0.13	98.75 ± 0.27
Drug loading (%)	9.32 ± 0.67	9.28 ± 0.58	9.23 ± 0.56
pH	5.78 ± 0.42	5.77 ± 0.12	5.76 ± 0.29
Transmittance (%)	96.80 ± 0.89	95.00 ± 0.76	97.03 ± 1.63
Dilution potential	>20 folds	>20 folds	>20 folds
Droplet size (nm)	206.00 ± 7.50	293.73 ± 6.27	227.00 ± 3.99
Polydispersity index	0.40 ± 0.04	0.51 ± 0.03	0.49 ± 0.01
ζ potential (mV)	−66.67 ± 0.42	−61.73 ± 3.96	−67.13 ± 0.06
Viscosity (cP)	33.13 ± 0.80	36.93 ± 0.25	38.03 ± 0.63

Data presented is the mean ± SD (n = 3).

**Table 2:** Results of kinetic stability of prepared LD-loaded nanoemulsions (S1–S3).

Batches	Centrifugation	Heat–Cool	Freeze–Thaw
S1	√	√	√
S2	√	√	√
S3	√	√	√

√: Stable (no phase separation, creaming, or changes in appearance).

**Table 3:** Gelation temperature and gelation time of tested Polaxamer 407 concentrations.

Polaxamer 407 Concentration %	Gelation Temperature (°C)	Gelation Time (min)
10	60	15
12	50	15
14	45	12
16	40	10
18	37	5
20	35	3

## Data Availability

The original contributions presented in this study are included in the article/supplementary material. Further inquiries can be directed to the corresponding author.

## Supplementary Materials

The following supporting information can be downloaded at: https://www.mdpi.com/article/10.3390/pharmaceutics17101355/s1, Table S1. Detailed statistical results of Cardiovascular safety studies; Figure S1. Observed particle size of the LD-loaded nanoemulsion (S2); Figure S2. Observed particle size of the LD-loaded nanoemulsion (S3); Figure S3. Observed zeta potential of the selected LD-loaded nanoemulsion (S2); Figure S4. Observed zeta potential of the selected LD-loaded nanoemulsion (S3); Figure S5. ECG baseline waveforms; Figure S6. ECG post-treatment waveforms.
