# Supplementary material for: Injectable In Situ Thermoreversible Gel Depot System of Lidocaine Nanoemulsion for Prolonged Anesthetic Activity in Dental and Operative Procedures
Source: Pharmaceutics. 2025 Oct 20;17(10):1355. doi: 10.3390/pharmaceutics17101355 (PMC12567530; doi:10.3390/pharmaceutics17101355)
Supplement: Supplementary file 1 [file pharmaceutics-17-01355-s001.zip › pharmaceutics-3901656-supplementary.pdf]

pharmaceutics

Supplementary Materials

Injectable In-Situ Thermoreversible Gel Depot System of Lidocaine Nanoemulsion for Prolonged Anesthetic Activity in Dental and Operative Procedures

Shery Jacob, Fathima Sheik Kather, Shakta Mani Satyam, Sai H. S. Boddu, Firas Assaf, Tasnem H. Abdelfattah Allam, and Anroop B. Nair

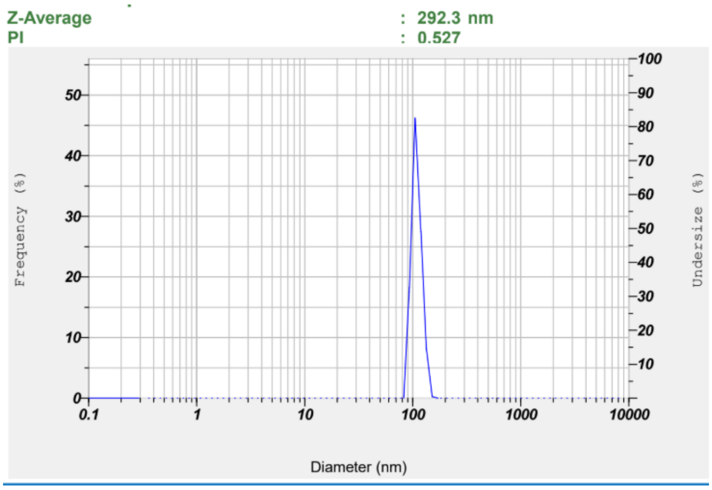

Figure S1. Observed particle size of the LD loaded nanoemulsion (S2).

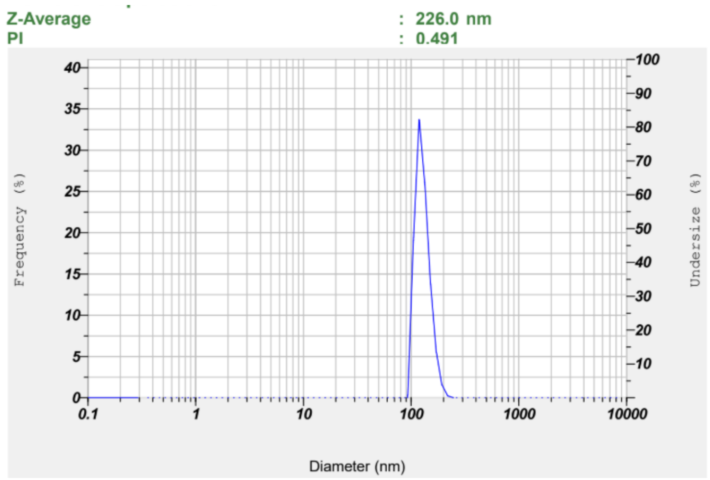

Figure S2. Observed particle size of the LD loaded nanoemulsion (S3).

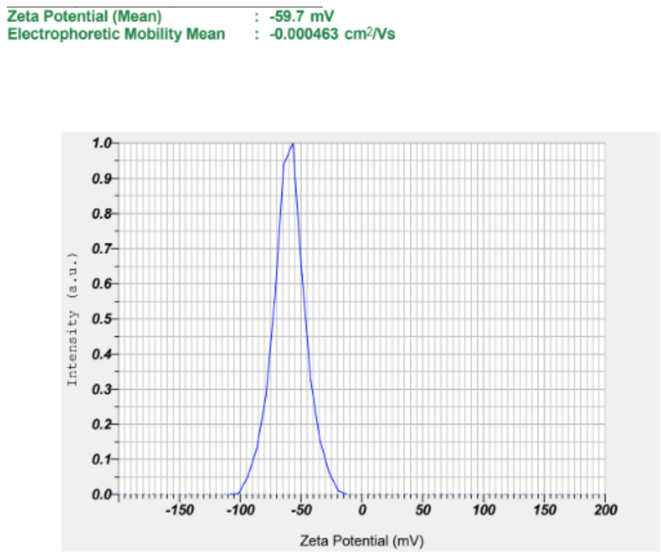

Figure S3. Observed zeta potential of the selected LD loaded nanoemulsion (S2).

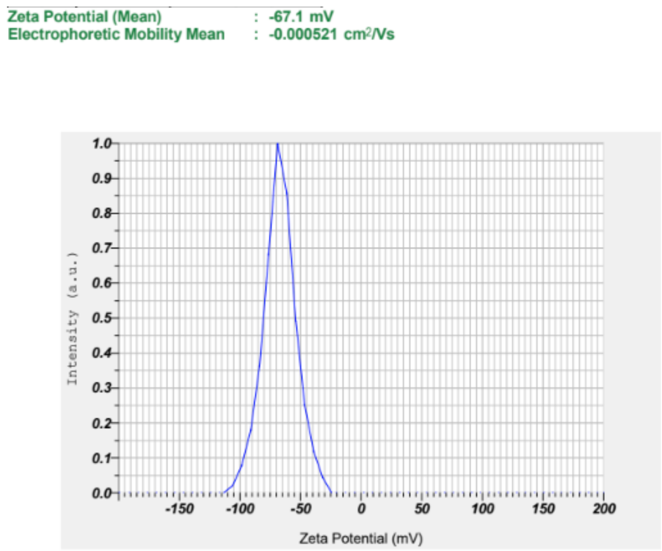

Figure S4. Observed zeta potential of the selected LD loaded nanoemulsion (S3).

Figures S5. ECG Baseline Waveforms

Blank Nanoemulgel group:

Rat 1:

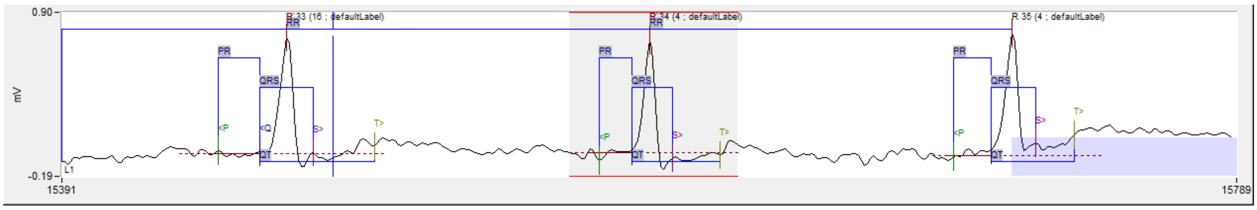

Rat 2:

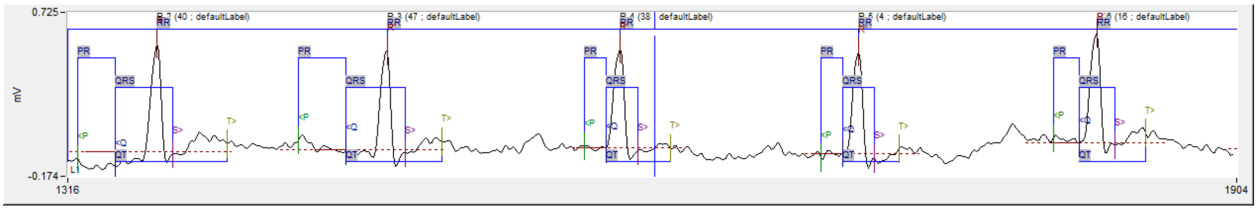

Rat 3:

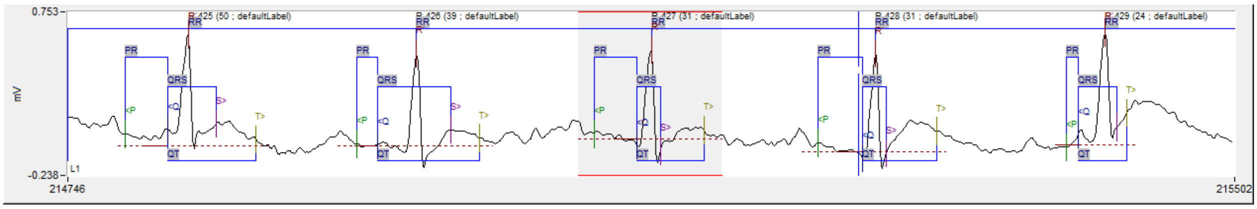

Rat 4:

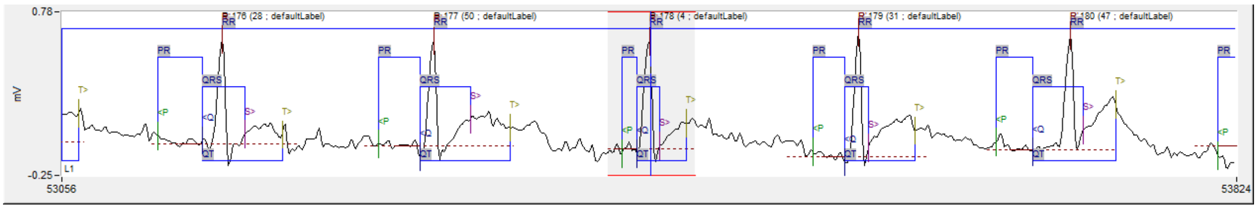

Rat 5:

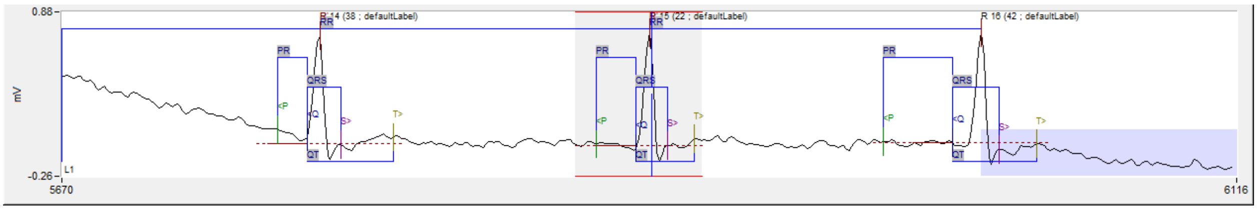

Rat 6:

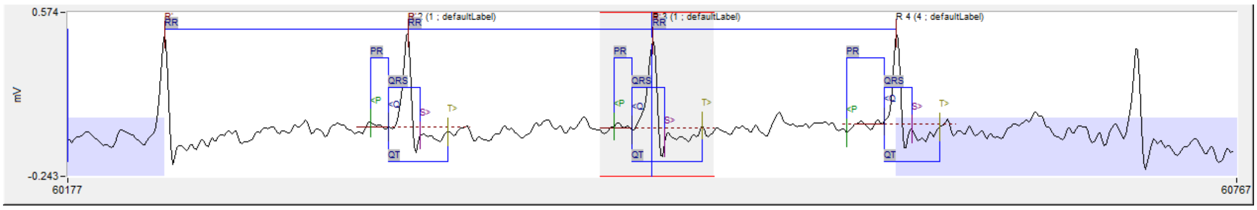

Standard LD 2% group:

Rat 1:

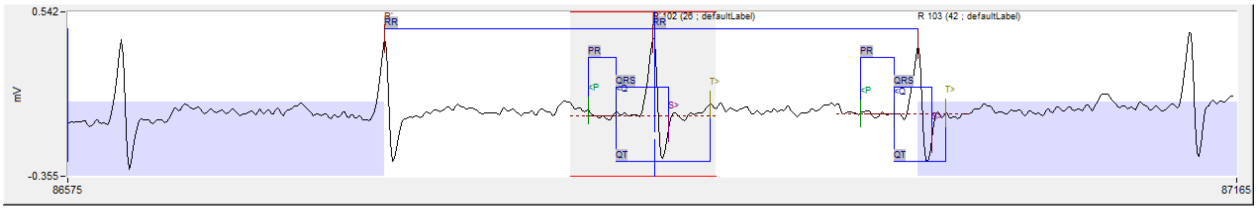

Rat 2:

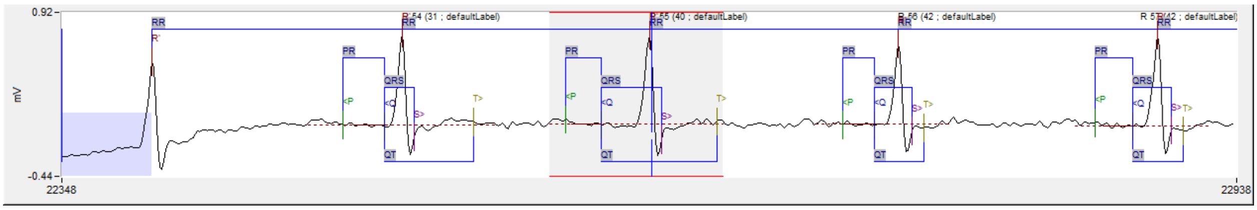

Rat 3:

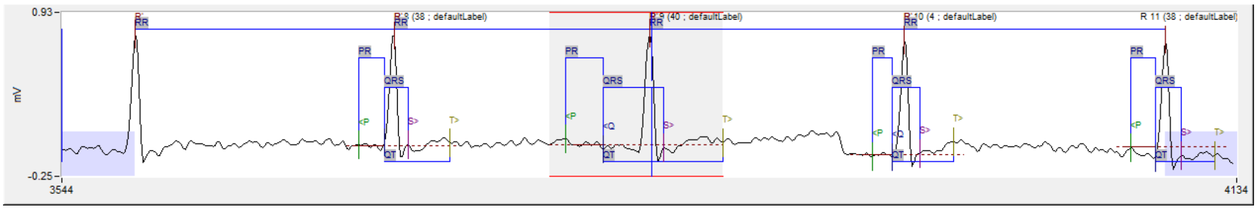

Rat 4:

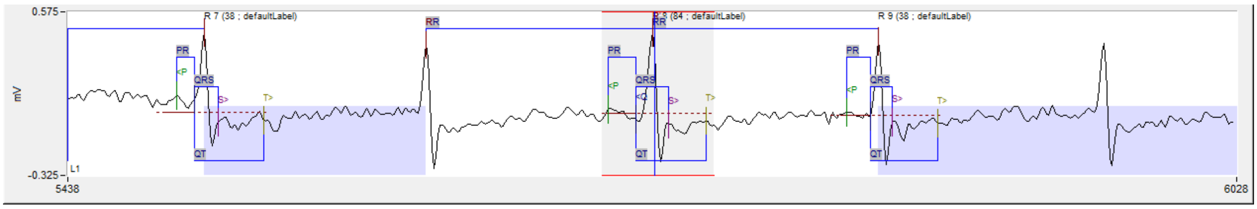

Rat 5:

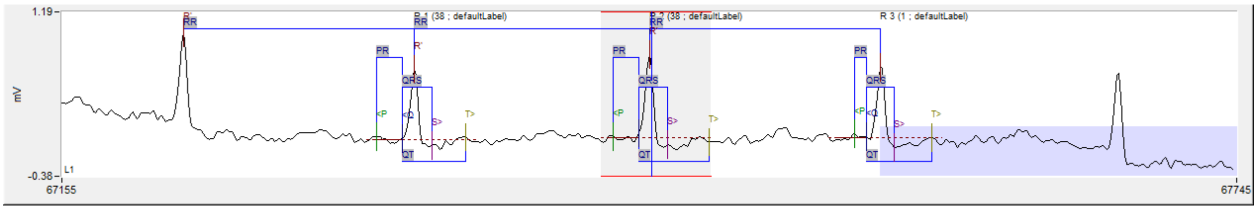

Rat 6:

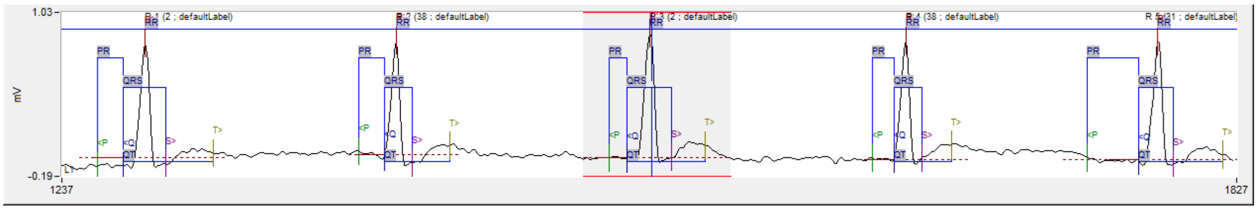

LD Nanoemulgel group:

Rat 1:

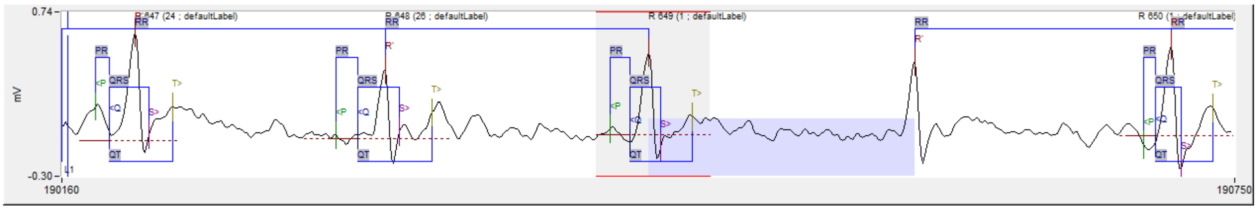

Rat 2:

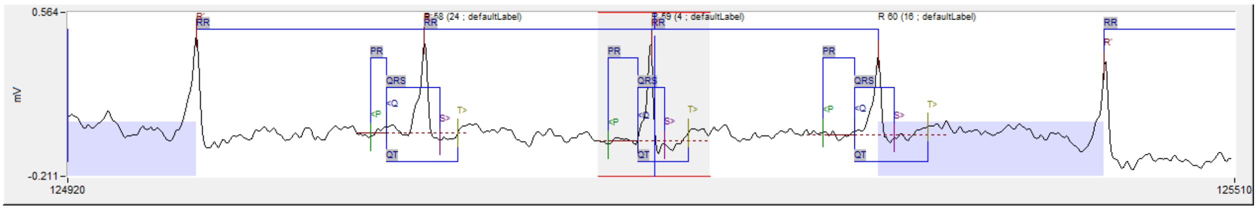

Rat 3:

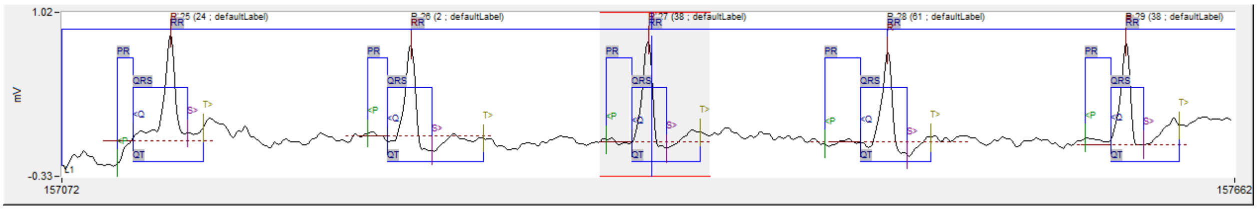

Rat 4:

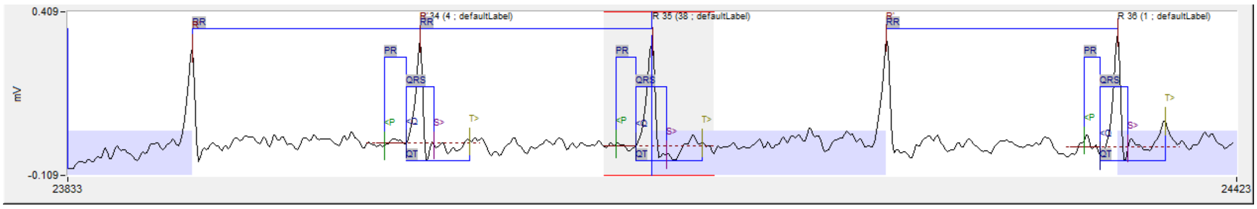

Rat 5:

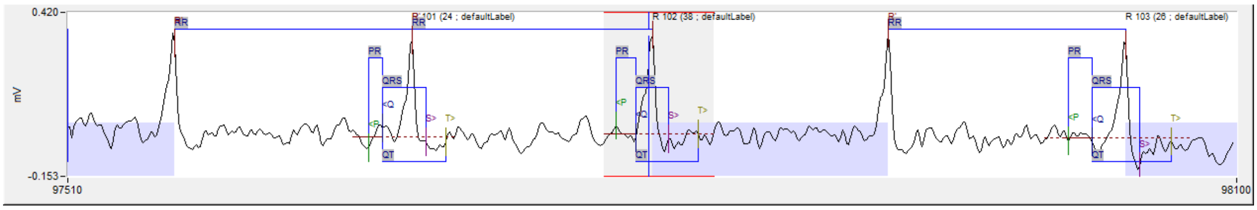

Rat 6:

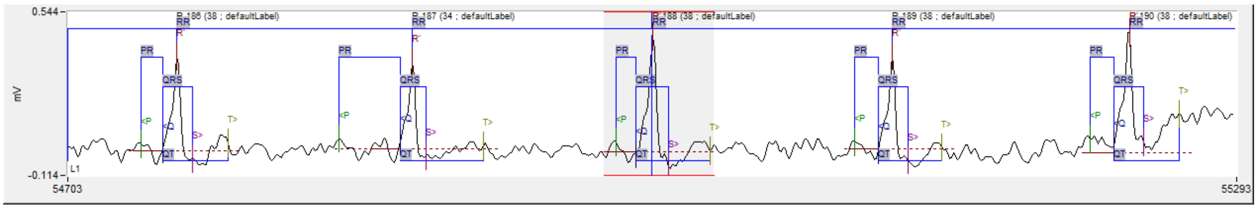

Figures S6. ECG Post-treatment Waveforms

Blank Nanoemulgel group:

Rat 1:

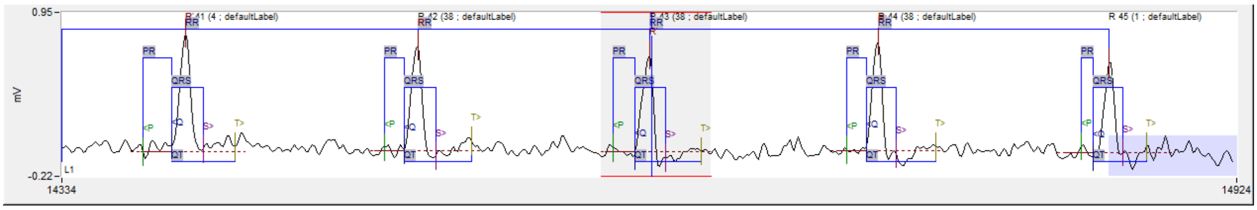

Rat 2:

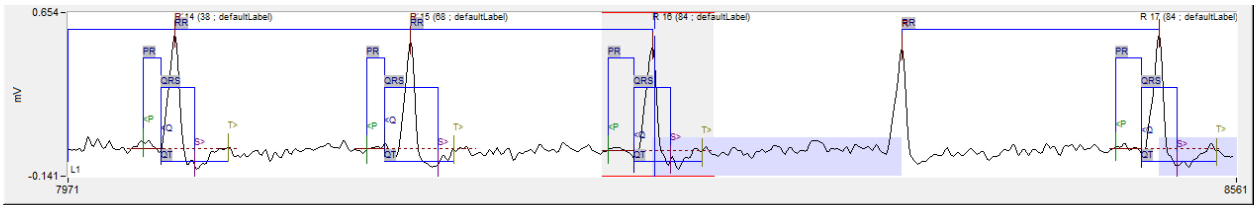

Rat 3:

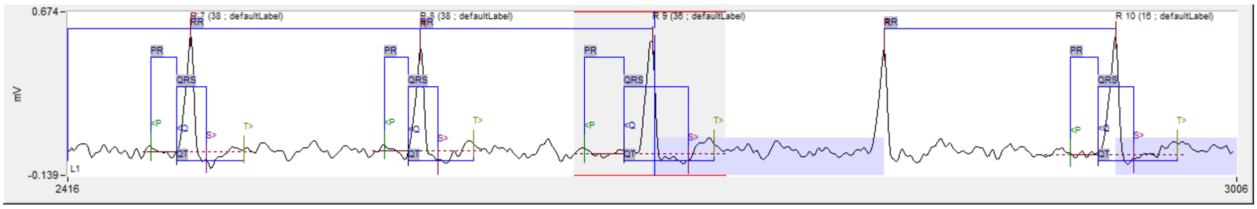

Rat 4:

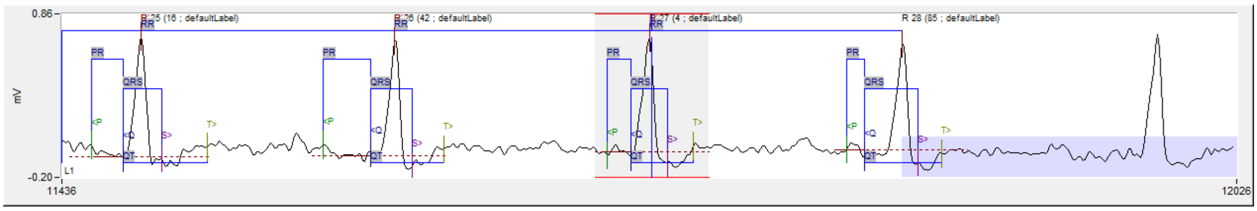

Rat 5:

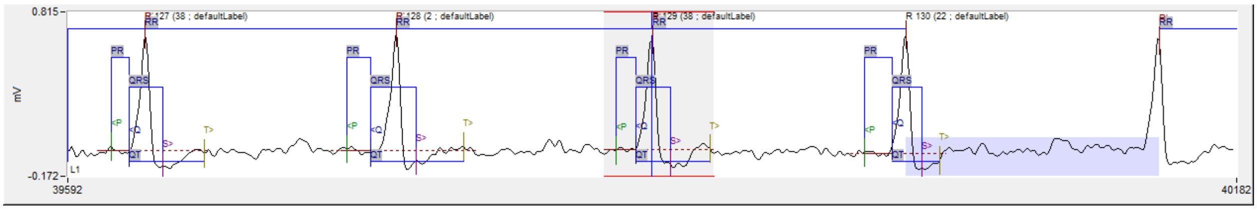

Rat 6:

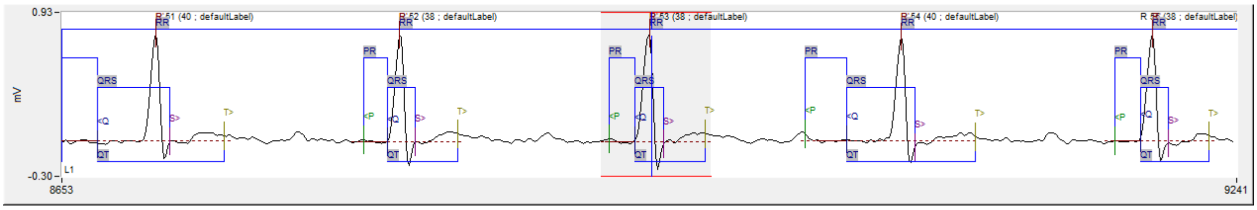

Standard LD 2% group:

Rat 1:

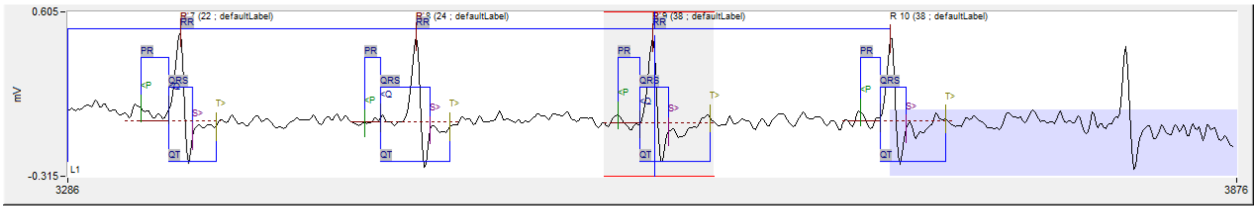

Rat 2:

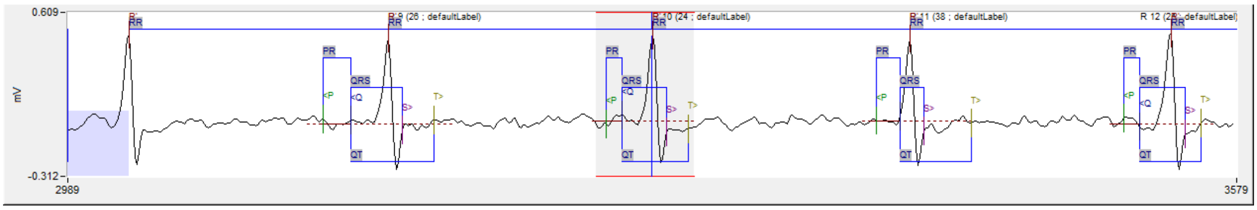

Rat 3:

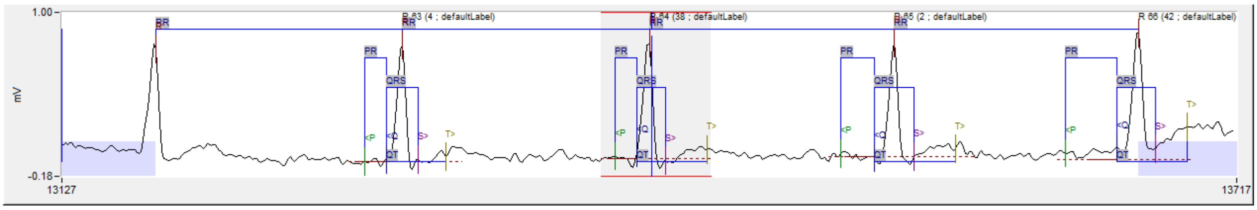

Rat 4:

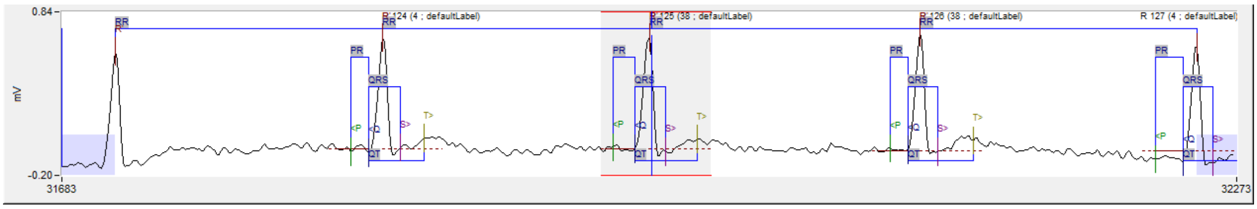

Rat 5:

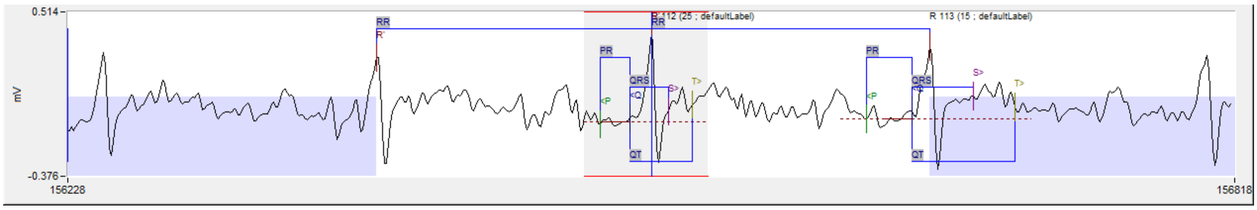

Rat 6:

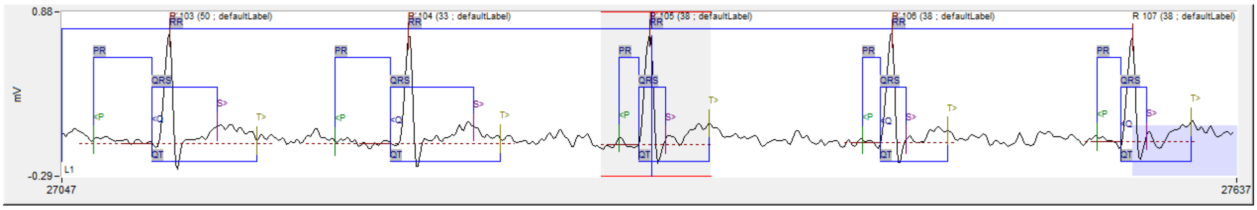

LD Nanoemulgel group:

Rat 1:

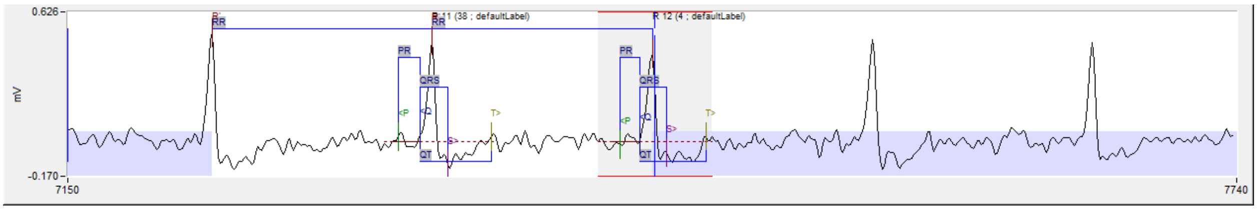

Rat 2:

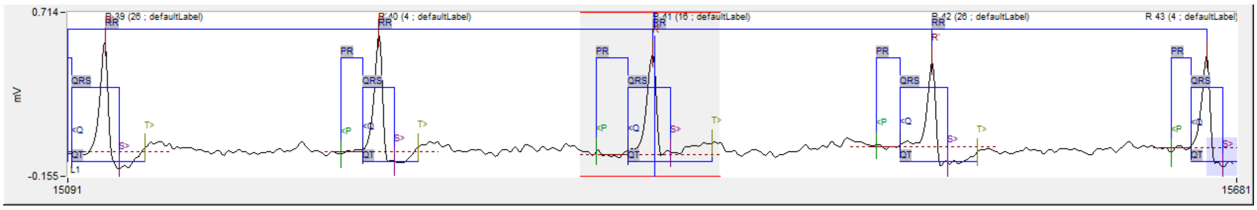

Rat 3:

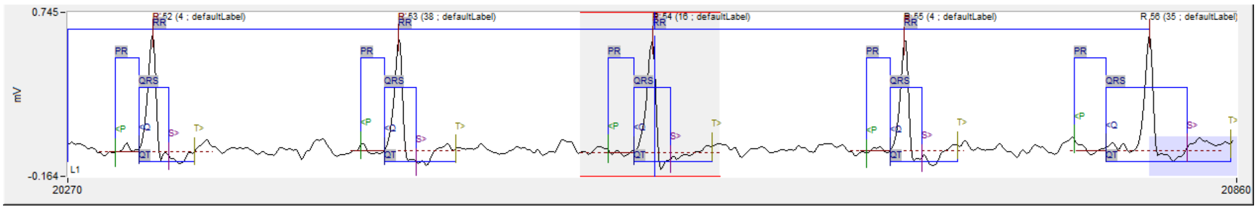

Rat 4:

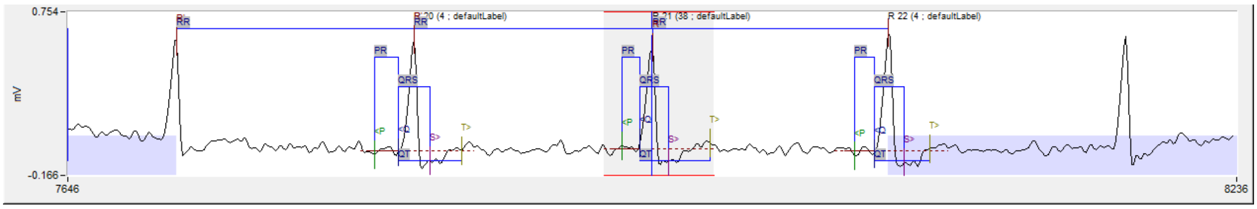

Rat 5:

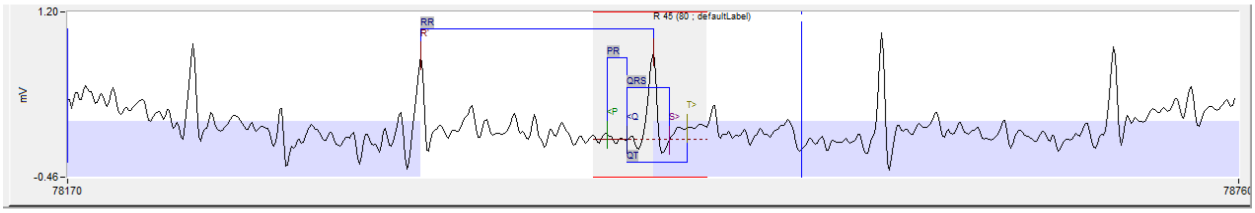

Rat 6:

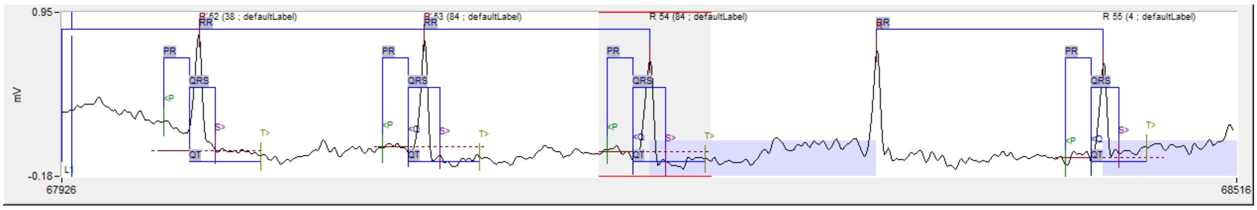

Table S1. Detailed statistical results of Cardiovascular safety studies.

		Groups			Significance (*p*)		
		Blank Nanoemulgel (NC)	Standard LD 2% (PC)	LD Nanoemulgel (Test)	NCvsPC	NCvsTest	PCvsTest
SBP	Pre_SBP	154.93±17.93	147.4±13.64	154.14±10.45	0.642	0.995	0.7
	Post_SBP	134.04±5.03	131.8±6.99	125.53±7.9	0.834	0.107	0.273
DBP	Pre_DBP	122.51±8.94	110.4±12.25	116.5±14.34	0.223	0.671	0.663
	Post_DBP	89.51±13.21	90.67±8.52	82.9±10.07	0.981	0.55	0.444
MAP	Pre_MAP	131.24±10.61	122.35±12.37	128.66±12.77	0.423	0.926	0.64
	Post_MAP	104.06±9.18	104.01±7.88	96.75±8.7	1	0.332	0.337
HR	Pre_HR	464.61±43.35	483.9±32.3	509.00±20.16	0.586	0.085	0.414
	Post_HR	477.58±27.71	461.29±36.78	482.37±49.36	0.753	0.975	0.626
PR Interval	Pre_PR	14.33±3.02	16.09±2.6	13.12±1.56	0.451	0.681	0.127
	Post_PR	14.93±1.68	15.14±2.17	13.77±1.42	0.977	0.514	0.4
QRS Complex	Pre_QRS	22.15±3.49	21.39±3.03	21.17±1.98	0.892	0.829	0.991
	Post_QRS	23.46±2.93	21.83±2.42	19.73±2.55	0.547	0.066	0.991
QT	Pre_QT	40.88±5.93	39.73±6.34	38.22±3.27	0.927	0.673	0.878
	Post_QT	41.73±4.84	40.0097±3.85	37.03±3.44	0.749	0.148	0.437
QTcB	Pre_QTcB	113.06±12.53	112.79±19.42	111.26±10.14	0.999	0.975	0.982
	Post_QTcB	117.44±12.32	110.67±9.88	104.46±6.9	0.483	0.093	0.54
QTcF	Pre_QTcF	80.53±9.77	79.65±13.33	77.91±6.91	0.988	0.901	0.955
	Post_QTcF	83.18±8.98	78.82±7.07	73.91±5.29	0.566	0.102	0.488
